# Genomic and Proteomic Studies on the Mode of Action of Oxaboroles against the African Trypanosome

**DOI:** 10.1371/journal.pntd.0004299

**Published:** 2015-12-18

**Authors:** Deuan C. Jones, Bernardo J. Foth, Michael D. Urbaniak, Stephen Patterson, Han B. Ong, Matthew Berriman, Alan H. Fairlamb

**Affiliations:** 1 School of Life Sciences, University of Dundee, Dundee, United Kingdom; 2 Parasite Genomics, Wellcome Trust Sanger Institute, Hinxton, Cambridge, United Kingdom; 3 Division of Biomedical and Life Sciences, Lancaster University, Lancaster, United Kingdom; Northeastern University, UNITED STATES

## Abstract

SCYX-7158, an oxaborole, is currently in Phase I clinical trials for the treatment of human African trypanosomiasis. Here we investigate possible modes of action against *Trypanosoma brucei* using orthogonal chemo-proteomic and genomic approaches. SILAC-based proteomic studies using an oxaborole analogue immobilised onto a resin was used either in competition with a soluble oxaborole or an immobilised inactive control to identify thirteen proteins common to both strategies. Cell-cycle analysis of cells incubated with sub-lethal concentrations of an oxaborole identified a subtle but significant accumulation of G2 and >G2 cells. Given the possibility of compromised DNA fidelity, we investigated long-term exposure of *T*. *brucei* to oxaboroles by generating resistant cell lines *in vitro*. Resistance proved more difficult to generate than for drugs currently used in the field, and in one of our three cell lines was unstable. Whole-genome sequencing of the resistant cell lines revealed single nucleotide polymorphisms in 66 genes and several large-scale genomic aberrations. The absence of a simple consistent mechanism among resistant cell lines and the diverse list of binding partners from the proteomic studies suggest a degree of polypharmacology that should reduce the risk of resistance to this compound class emerging in the field. The combined genetic and chemical biology approaches have provided lists of candidates to be investigated for more detailed information on the mode of action of this promising new drug class.

## Author Summary

The mode of action of a new class of boron-containing chemicals (the oxaboroles), currently under development for the treatment of human African trypanosomiasis, is unknown. Here we identify a number of potential candidate proteins that could be involved either in the mode of action of these compounds or in the mechanism of resistance. This information could prove critical in protecting the compounds against resistance emerging in the field as well as opening up new avenues for drug discovery.

## Introduction

Human African trypanosomiasis (HAT) is caused by two subspecies of the unicellular parasite *Trypanosoma brucei*, an infection which is transmitted by the bite of a tsetse fly. HAT progresses through a haemo-lymphatic stage into a meningo-encephalitic stage [1] and has a fatality rate close to 100% if left untreated [2]. The disease is also a key factor in maintaining the poverty cycle, and patients are often discriminated against or abandoned [3]. The reporting of new cases of HAT has fallen to below 7,000 in 2011 [4]. However, the disease has previously resurged from even lower levels in the 1980s and 1990s [5]. Current estimates place 70 million people at risk with more than 5 million living in areas of high or very high risk for contracting HAT [4].

*T*. *brucei gambiense* is responsible for around 98% of reported cases [5], and has been targeted by the World Health Organization for elimination by 2020. However, elimination of *T*. *brucei rhodesiense*, which has epidemic potential, is not feasible due to its animal reservoir [5]. The tsetse fly vector also presents significant risks to disease control in that climate change may allow the vector to invade new geographical regions [6], and sexual recombination, which occurs within the vector, could allow rapid transfer of drug resistance and virulence factors [7]. Hence, whilst improvements in control have been achieved, there are several risk factors that could lead to resurgence of the disease [8,9].

Existing drugs are highly unsatisfactory due to toxicity, mode of administration and efficacy [8]. The ease of developing resistance to both components of the nifurtimox / eflornithine combination therapy (NECT, the newest treatment to enter the clinic) [10] is also a major concern [11,12]. Moreover, due to its status as a neglected disease of declining incidence, the current drug discovery pipeline for HAT is far from robust [13]. Thus, development of new drugs remains a critical task.

Recent advances have included the entry of fexinidazole into phase II/III trials against HAT (ongoing) [14] and the identification of oxaboroles as a class of compounds active against *T*. *brucei* by a collaboration between the Drugs for Neglected Disease initiative, Anacor Pharmaceuticals and SCYNEXIS [15]. One member of this class, SCYX-7158, shown to be effective in the meningo-encephalitic stage of HAT [16], entered phase I clinical trials in March 2012 and studies, including safety profiling, are ongoing (DNDI diseases and projects portfolio accessed 14/08/15 www.dndi.org/diseases-projects/portfolio/oxaborole-scyx-7158]).

Oxaborole compounds have been demonstrated to act via inhibition of leucyl RNA synthetase as anti-pneumococcal agents [17] and anti-fungal agents [18]. They can also form adducts with *cis*-diols in sugars and have been shown to inhibit other enzymes such as phosphodiesterases, β-lactamases and kinases (see review [19]). However, the mode of action against African trypanosomes has not been determined. This information would inform the selection of appropriate partner compounds to protect against resistance, and could also open up novel areas of drug discovery.

Our objective was to apply genomic sequencing and chemo-proteomic approaches [20] to facilitate mode of action studies on the oxaborole series, an approach which has been successful with other antitrypanosomal compounds [21,22]. Here, we report the use of two orthogonal methods (proteomic studies using affinity chromatography and stable isotope labelling by amino acids in cell culture [SILAC]; and whole genome sequencing of sensitive and drug-resistant cell lines) to produce lists of candidate targets for oxaborole compounds that will be pursued in future work. Our experiments suggest a high level of polypharmacology that could protect the oxaborole class from resistance emerging in the field.

## Materials and Methods

### Chemicals

All chemicals were obtained from Sigma-Aldrich (Gillingham, UK) unless otherwise indicated. Amino acids for SILAC labelling (4,4,5,5-D4 L-Lysine and U-13C6 L-Arginine) were obtained from CK Gas Products (Hampshire, UK). PBS was formulated in-house. Foetal bovine serum for HMI9T was obtained from PAA Laboratories (Yeovil, UK), dialysed foetal bovine serum for SILAC-labelling was obtained from Life Technologies (Paisley, UK). IMDM for SILAC–labelling (lacking Arginine and Lysine) was obtained from Thermo Scientific (Basingstoke, UK). The cOmplete EDTA-free protease inhibitor cocktail was obtained from Roche Diagnostics (West Sussex, UK).

### Cell culture

Bloodstream-form *T*. *brucei* ‘single marker’ cells [23] were cultured at 37°C with 5% CO_2_ in HMI9T medium [24]. Cells were counted using a CasyCounter model TT (Roche Innovatis, Reutlingen) and maintained at densities below 5×10^6^ ml^−1^, sub-culturing as necessary. EC_50_ determinations were carried out using a resazurin-based assay, and means weighted to the standard error calculated as previously described [25,26].

SILAC-labelling was carried out using an adapted HMI11 [27]; *T*. *brucei* log-phase cells in HMI9T medium were washed in PBS and seeded at 1×10^4^ ml^−1^ into HMI11-SILAC + R_6_K_4_ as described previously [28]. Following 3 days growth to ~1×10^6^ ml^−1^, cells were harvested by centrifugation, washed in ice-cold PBS, and resuspended in PBS at 1.25×10^9^ ml^−1^. Four parts cell suspension was mixed with one part 5× lysis buffer (25% glycerol; 30 mM MgCl_2_; 4% IGEPAL CA-630 (octylphenoxy poly(ethyleneoxy)ethanol); 5 mM DTT), cOmplete protease inhibitor cocktail added to 1× concentration and the sample freeze-thawed three times. DNase-I was added to 1 μg ml^−1^, the mixture incubated on ice for 5 min, vortexed for 10 s and clarified by centrifugation at 20,000 *g* for 1 h at 4°C. The supernatant was divided into 500 μl aliquots, adjusted to 5 mg ml^−1^ with 1 × lysis buffer, snap frozen, and stored at -80^°^C prior to subsequent processing.

### Synthesis of oxaboroles and immobilisation to paramagnetic beads

SCYX-6759 and oxaborole-1 were prepared using previously published methodology [29]. Full experimental details for the synthesis of the oxaboroles utilised in this study are given in the Supporting Information (S1 Text). Beads derivatised with an oxaborole, or control compound were prepared as follows. The storage solvent was removed from commercial NHS functionalised magnetic beads (Thermo Scientific) and the beads washed and resuspended in anhydrous DMSO (150 μl [mg resin]^−1^). The amine-containing compound (7 nmol [mg resin]^−1^) and DIPEA (14 nmol [mg resin]^−1^) were then added and the resin gently agitated for 24 h at room temperature. After which the reaction solvent was removed, and the beads washed and resuspended in anhydrous DMSO (150 μl [mg resin]^−1^). Ethanolamine (70 nmol [mg resin]^−1^) and DIPEA (70 nmol [mg resin]^−1^) were then added and the resin gently agitated for 24 h at room temperature. The reaction solvent was then removed and the resin washed with DMAc, prior to storage in the same solvent. Note, the incubation step with an amine-containing compound is omitted when preparing ‘blank’ ethanolamine-capped beads.

### Chemical proteomic profiling

**Fig 1:**
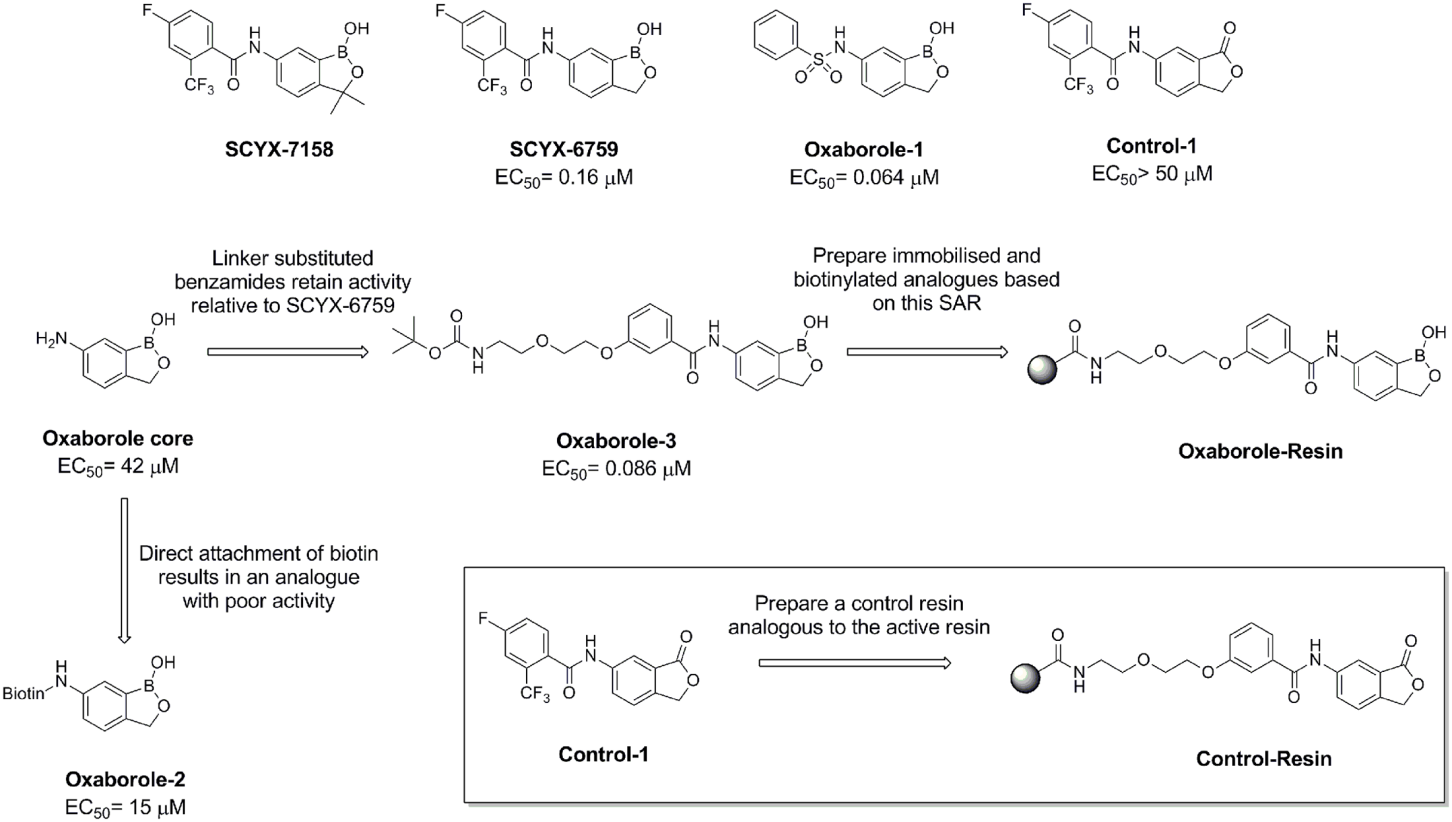
Structure of oxaboroles and affinity chromatography resins.

Lysates were pre-cleared by incubation with ethanolamine-capped paramagnetic beads (0.2 mg) for 30 min at 4°C, after which the supernatant was transferred to a new sample tube along with a 50 μl wash. For competition experiments, oxaborole-1 in DMSO (1 μM final concentration) or a DMSO control was added (0.5% DMSO final) and incubated with mixing for 30 min at 4°C. Subsequently, 0.2 mg of oxaborole-resin (Fig 1) was added to each sample and incubated for a further 60 min at 4°C. The beads were isolated using a magnet, washed twice with lysis buffer and united into a single sample tube. The beads were further washed three times with PBS, and bead-bound proteins were eluted with NuPAGE LDS buffer (Invitrogen) containing 50 mM DTT for 5 min at 95°C. For comparison of the oxaborole resin and control resin, pulldowns were performed in a similar manner in the absence of soluble compound.

### Polyacrylamide gel electrophoresis

Eluted samples were subjected to electrophoresis on a NuPAGE bis-Tris 10% acrylamide gel until the dye front had entered about 1 cm into the gel. The proteins were stained with InstantBlue (Expedeon), and the entire stained area excised and subjected to in-gel digestion for 18 h at 37°C with 12.5 μg ml^−1^ trypsin gold (Promega) in 10 mM NH_4_HCO_3_, 10% MeCN. Tryptic peptides were recovered in 45% MeCN, 1% formic acid and lyophilized prior to analysis.

### Mass spectrometry data acquisition and processing

Liquid chromatography tandem mass spectrometry was performed by the Fingerprints Proteomic Facility at the University of Dundee, as described previously [28]. Data was processed using MaxQuant [30] version 1.3.0.5 which incorporates the Andromeda search engine [31]. Proteins were identified by searching a protein sequence database containing *T*. *brucei brucei* 927 annotated proteins (Version 4.0, downloaded from TriTrypDB [32], http://www.tritrypdb.org/) supplemented with the VSG221 sequence and frequently observed contaminants (porcine trypsin, bovine serum albumin and mammalian keratins) that contains a total of 10,081 protein sequences. Search parameters specified an MS tolerance of 6 ppm, an MS/MS tolerance at 0.5 Da and full trypsin specificity, allowing for up to two missed cleavages. Carbamidomethylation of cysteine was set as a fixed modification and oxidation of methionine residues, *N*-terminal protein acetylation and *N*-pyroglutamate were allowed as variable modifications. Peptides were required to be at least 7 amino acids in length and a MaxQuant score >5, with false discovery rates (FDRs) of 0.01 calculated at the levels of peptides, proteins and modification sites based on the number of hits against the reversed sequence database. SILAC ratios were calculated using only peptides that could be uniquely mapped to a given protein group, and required a minimum of two SILAC pairs. To account for any errors in the counting of the number of cell numbers mixed, the distribution of SILAC ratios was normalised within MaxQuant at the peptide level so that the median of log_2_ ratios is zero [30]. Data were visualized using Perseus 1.3.0.4 (www.perseus-framework.org) and further information on the identified proteins was obtained from TriTrypDB [32] (http://www.tritrypdb.org).

### Cell-cycle analysis

*T*. *brucei* cultures (50 ml) were seeded at 5×10^5^ ml^−1^ in the presence of 225 nM Oxaborole-1 (5× EC_50_). Samples (4 ml) were taken at 0, 14, 20 and 28 h, collected by centrifugation at 850 *g* for 10 min and processed essentially as previously described [33]. Briefly, cells were washed in 1 ml PBS containing 1% FBS, the supernatant was removed and cells resuspended in the residual volume. Cells were fixed with 1 ml ice cold 70% ethanol, adjusted to 5×10^5^ ml^−1^ and washed twice with PBS containing 1% FBS. Cells were resuspended in 400 μl staining solution (PBS containing 1% FBS, 50 μg ml^−1^ propidium iodide, 50 μg ml^−1^ RNase A), stained for 20 min at room temperature before being analysed by flow cytometry as previously described [33].

### Generation of oxaborole-resistant cell lines

Oxaborole-resistant *T*. *brucei* were generated in three independent flasks by sub-culturing in the presence of increasing concentrations of Oxaborole-1. Beginning at the sub-lethal concentration of 20 nM, the process was continued until the cells were growing in 500 nM (~10 × original EC_50_). Throughout the process, increasing concentrations of Oxaborole-1 were attempted once the cells were displaying cell growth and motility similar to a control grown in the absence of the drug. After 180 days, cells were cloned by limiting dilution in the presence of 500 nM Oxaborole-1 to yield independent clones from each of the three flasks.

The three resistant clones were diluted 1000-fold into media without Oxaborole-1 and sub-cultured as necessary over a two month period. EC_50_ determinations were carried out to indicate whether resistance had been maintained in the absence of exposure to Oxaborole-1.

### Genomic sequencing and analysis

Genomic DNA was prepared from five *T*. *brucei* lines, i.e. the parental clone Lister 427 (SM), three oxaborole-resistant clones (clone 1, 2, and 3), and a drug-resistance revertant clone (clone 1R). For each sample, 0.6–2 μg of genomic DNA was used to produce standard Illumina libraries of 400–600 base pairs (bp) [34]. Sequencing was carried out on an Illumina HiSeq 2000 sequencer according to the manufacturer’s standard sequencing protocol and yielded 22.8–29.6 million reads of 100 bp length per library. These data sets represented a nominal sequencing coverage of the *T*. *brucei* genome (35Mb) of approximately 65.2- to 84.6-fold. The Illumina data were aligned against the *T*. *brucei brucei* TREU927 reference genome [35] assembly using SMALT v0.7.4 (http://www.sanger.ac.uk/resources/software/smalt/). For variant calling, the alignment was run employing an exhaustive search (-x) and with parameters wordlen = 13 (-k), skipstep = 1 (-s), minscor = 0.8 (-m), and insertmax = 1000 (-i). To assess relative read coverage and copy number variations (CNVs), the alignment runs were repeated using the above parameters with repetitive mapping (-r) enabled which results in read pairs with multiple equally good alignment positions being aligned to one of these locations at random. Variants were called using SAMtools v0.1.19 mpileup (-Q 15 for baseQ/BAQ filtering) and BCFtools [36]. To exclude the hypervariable subtelomeric regions, only variants found in the following chromosomal core regions were included in the downstream analyses: Tb927_01_v4:202,695–988,120; Tb927_02_v4:259,723–1,161,408; Tb927_03_v4:146,614–1,602,829; Tb927_04_v4:80,380–1,467,268; Tb927_05_v4:72,088–1,366,595; Tb927_06_v4:111,409–1,414,033; Tb927_07_v4:26,571–2,177,541; Tb927_08_v4:135,192–2,476,033; Tb927_09_v4:325,850–2,394,987; Tb927_10_v5:55,698–3,993,940; Tb927_11_01_v4:36,585–4,482,610. SNP calls were further filtered for all of the following: for a minimum of 8 "high-quality" base calls ("DP4"); for a minimum phred-scale QUAL score of 20; for a maximum phred-scale likelihood of the best genotype call of 5 ("PL1"); for a minimum phred-scale likelihood of the second best genotype call of 10 ("PL2"); for a minimum strand bias P-value of 0.01 (first of "PV4"); for a maximum ratio of conflicting base calls for homozygous genotypes of 5%; and, for positions with a minimum and maximum read depth of three times the median read depth observed for that chromosome: for a minimum mapping quality of 20; and for a minimum distance of 10 nucleotides from the nearest INDEL call. Files in Variant Call Format (VCF) listing all 206,417 genomic positions at which any one of the five sequenced parasite lines had a variant call are available in the supplementary material (S1–S5 Datasets). The illustrations showing the location of genes along chromosomal regions in Figure CNVs was generated using Web-Artemis (http://www.genedb.org/web-artemis/).

### Accession numbers

The raw sequence data are available under the following accession numbers at the European Nucleotide Archive (http://www.ebi.ac.uk/ena): parent: ERS136142; resistant clone 2: ERS136134; resistant clone 3: ERS136135; resistant clone 1: ERS136145; revertant clone of clone 1: ERS136137. The raw and processed mass spectrometry data have been deposited with the ProteomeXchange Consortium [37] (http://www.proteomexchange.org/) via the PRIDE partner repository under the identifier PXD002848.

## Results and Discussion

### Chemical synthesis / immobilisation

The synthesis of SCYX-6759 and Oxaborole-1 was readily achieved using published procedures [29]. Initial attempts to immobilise the oxaborole scaffold involved the direct attachment of biotin (for use in conjunction with a streptavidin resin) to the aniline functionality to give Oxaborole-2 (Fig 1 and S1 Fig). However, subsequent biological assay demonstrated that Oxaborole-2 was only weakly active against *T*. *brucei* (EC_50_ 15 μM) compared to SCYX-6759 (EC_50_ 0.16 μM), Oxaborole-1 (EC_50_ 0.064 μM) or SCYX-7158 (EC_50_ 0.79 μM, data from [16,38]). Therefore, a small number of analogues retaining the benzamide functionality of SCYX-6759 and SCYX-7158 were prepared (one of which is shown in S2 Fig). Oxaborole-3, which contains a polyethyleneglycol linker in the *meta* position of the benzamide was found to retain activity in the bloodstream form *T*. *brucei* assay (EC_50_ 0.086 μM). The carbamate protecting group of Oxaborole-3 was subsequently removed and the resultant primary amine reacted to prepare an amide of biotin (Oxaborole-Biotin, S2 Fig), which in this case was found to be bioactive (EC_50_ 0.40 μM). An analogue of SCYX-6759, where the oxaborole bicycle was replaced with a phthalide bicycle (Control-1) was prepared and found to be inactive against *T*. *brucei*. Therefore, a control biotin conjugate (Control-Biotin) was prepared in an analogous fashion to Oxaborole-Biotin (S3 Fig). Pilot chemical proteomics studies suggested that the use of a biotin-conjugate/streptavidin bead system was sub-optimal. As a result, the linker containing oxaborole and control analogues were instead attached to paramagnetic beads via an amide linkage to give an Oxaborole-Resin and a Control-Resin respectively (Fig 1 and S2 and S3 Figs).

### Chemical proteomic profiling

In order to directly profile the proteins that bound to the Oxaborole-Resin, chemical proteomic profiling was undertaken using two orthogonal strategies that utilised SILAC quantitation to eliminate non-specific binding proteins. In these experiments parasites are grown in identical media where one contains “light” and the other “heavy” amino acid isotopes (in this case arginine and lysine). After several rounds of cell division, the two populations are identical except for the differential labelling of the proteome with either light or heavy isotopes. After undergoing differential processing, the two samples are combined and the ratio of heavy to light peptide from each individual protein determined by mass spectrometry. Proteins that are specifically enriched by the differential treatment will have a heavy to light ratio not equal to 1, whereas proteins affected equally with have a ratio = 1 (or binary logarithm of 1 = zero).

**Fig 2:**
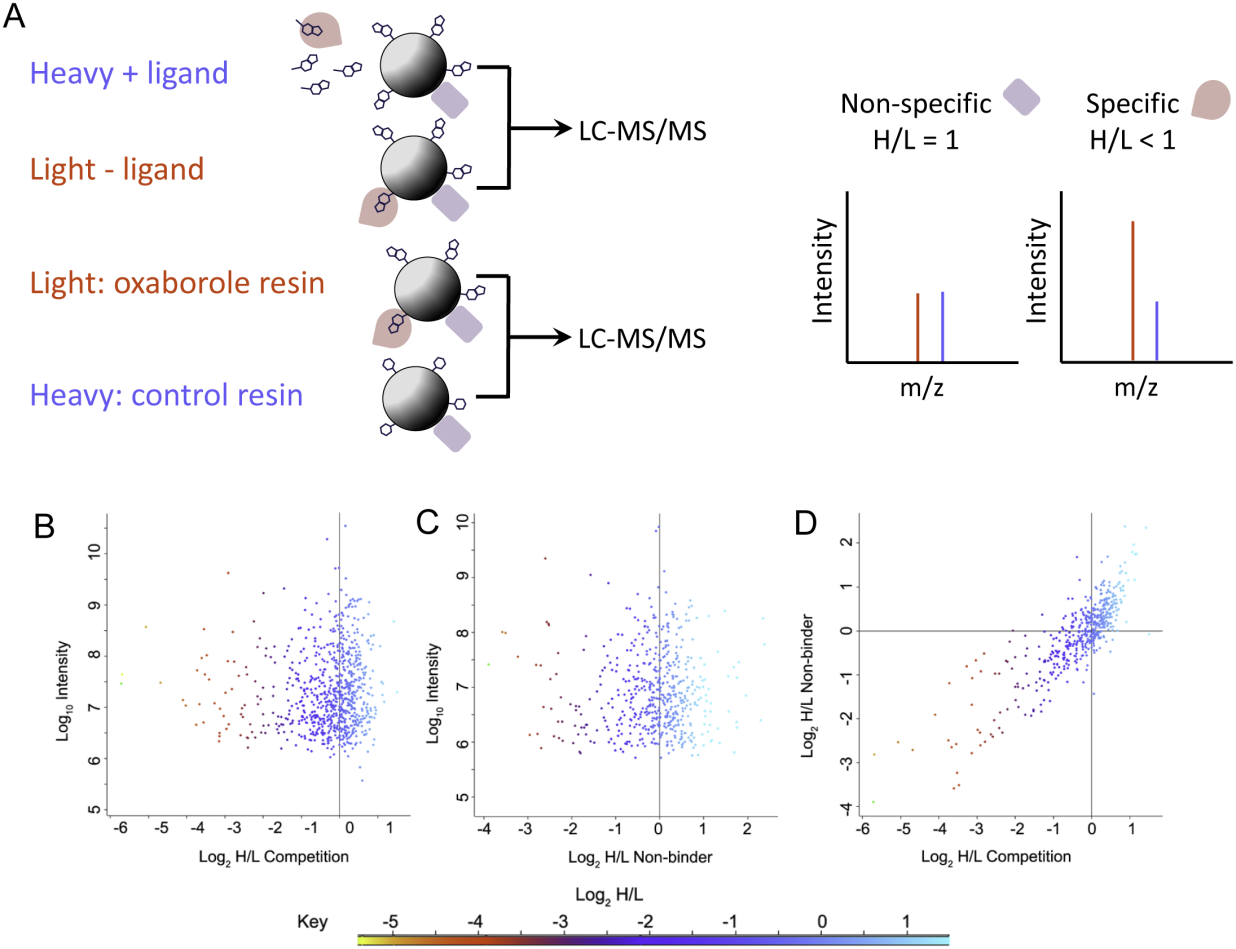
Quantitative chemical proteomic profiling of oxaborole targets. **A**–Schematic of proteomic profiling strategies representing ligand competition experiment (top) and resin comparison experiment (bottom). **B**–Drug competition experiment. SILAC-labelled *T*. *brucei* cell lysates were incubated with 10 μM Oxaborole-1 (heavy label) or DMSO control (light label), prior to enrichment of protein with Oxaborole-Resin. **C**–Comparison of analogue-bound resin. Proteins were enriched from SILAC labelled *T*. *brucei* cell by Oxaborole-Resin (light label) or Control-Resin (heavy label). **D**–Correlation of the proteins quantified by the two strategies. A colour key is included to show the range of enrichment of identified proteins.

In the first strategy, the profile of proteins from *T*. *brucei* cell lysates that bind the beads in the presence (heavy label) or absence (light label) of soluble inhibitor was quantified (Fig 2). Non-specific binders will be unaffected by the presence of soluble compound, thus will produce an equal heavy to light ratio (log_2_ H/L = 0). In contrast, the specific binders will bind Oxaborole-1 in the pre-incubation step, making them unavailable to bind to the immobilised oxaborole, resulting in a low heavy to light ratio (log_2_ H/L < 0) (Fig 2A, upper pair).

In the second strategy, an inactive Control-Resin was prepared (Fig 1 and S3 Fig). The profile of proteins from *T*. *brucei* cell lysates that bind the Control-Resin (heavy label) or Oxaborole-Resin (light label) can then be quantified (Fig 2). Proteins that bind non-specifically, or whose binding is not related to activity, produce an equal heavy to light ratio (log_2_ H/L = 0), whereas proteins whose binding correlates with activity will have a low heavy to light ratio (log_2_ H/L < 0) (Fig 2A, lower pair).

The results of the two orthogonal strategies are shown in Fig 2 and Table 1, with the full data presented in the supplementary material (S1 Table). The binding of a subset of proteins was prevented by the presence of the soluble compound (Fig 2B), with 42 proteins displaying greater than a four-fold reduction in binding (log_2_ H/L < -2). In the orthogonal strategy, a subset of 24 proteins displayed greater than a four-fold reduction in binding (log_2_ H/L < -2) to the Control-Resin compared to the Oxaborole-Resin (Fig 2C). Comparing the profile of the proteins quantified in both experiments revealed a strong correlation (Pearson 0.841) between the proteins that are displaced by Oxaborole-1 and those that bind only the Oxaborole-Resin and not the Control-Resin (Fig 2D). The 14 proteins that display greater than four-fold selectivity in each experiment, presented in Table 1, can be considered to be specific targets of Oxaborole-1. The number of specific targets identified and the lack of discernible commonality strongly suggest that oxaboroles display considerable polypharmacology, and provide too great a number to investigate systematically as individual targets.

**Table 1:** Specific binding partners of the Oxaborole-Resin revealed by proteomic profiling.

GeneDB IDs	Product	Function	Ligand competition	Resin comparison
			Log_2_ H/L	Log_2_ H/L
Tb927.9.6870*	RNA-binding protein, putative (RBSR1)	Possible pre-mRNA splicing	-5.68	-2.98
Tb927.10.3200*	U2 splicing auxiliary factor, putative (U2AF35)	pre-mRNA splicing	-5.06	-2.53
Tb927.8.3060*	cytosolic leucyl aminopeptidase, putative,metallo-peptidase, Clan MF, Family M17	proteolysis	-4.68	-2.70
Tb927.8.4810	prohibitin 1 (PHB1)	mitochondrial biogenesis	-3.64	-3.57
Tb927.10.2890*; Tb11.v5.0650	enolase	glycolysis	-3.54	-2.57
	enolase, putative			
Tb927.9.8720	fructose-1,6-bisphosphatase (FBPase)	gluconeogenesis	-3.52	-3.22
Tb927.6.1570*	2-hydroxy-3-oxopropionate reductase, putative	valine, leucine and isoleucine degradation	-3.14	-2.77
Tb927.11.11250	cytosolic malate dehydrogenase (cMDH)	glycolysis	-2.98	-2.26
Tb927.4.2080*	C2 domain containing protein (CC2D)	flagellum biosynthesis	-2.96	-2.29
Tb927.6.4280*; Tb927.6.4300	glyceraldehyde 3-phosphate dehydrogenase, glycosomal (GAPDH)	glycolysis	-2.90	-2.59
Tb927.11.11680*	2-oxoglutarate dehydrogenase E2 component, putative	tricarboxylic acid cycle	-2.79	-2.51
Tb927.4.1890	hypothetical protein, conserved		-2.55	-2.02
Tb927.7.1310*	hypothetical protein, conserved		-2.42	-2.21

*Abnormal RIT-SEQ growth phenotype in bloodstream forms at either day 3 or day 6 or both [39].

### Cell-cycle analysis

**Fig 3:**
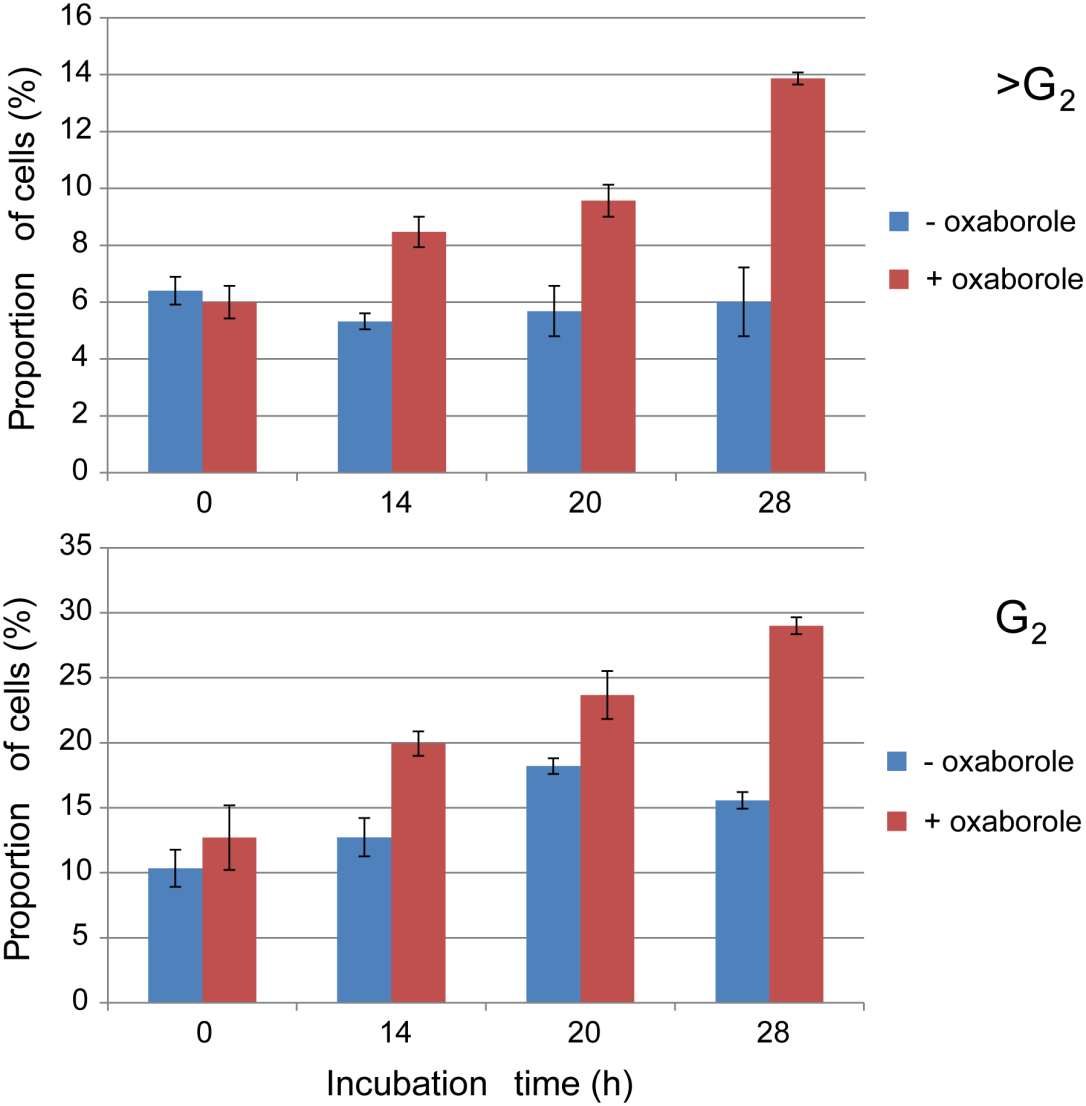
Cell cycle analysis following treatment with Oxaborole-1.

FACS analysis of *T*. *brucei* cells incubated with 225 nM Oxaborole-1 (5× EC_50_) indicated a statistically significant increase in the proportion of G_2_ and >G_2_ cells compared to the untreated control (Fig 3). These increases probably result from re-replication of DNA in the absence of cytokinesis. DNA re-replication has been seen in a variety of mutant *T*. *brucei* cell lines; however, it is possible that the cytokinesis defect is an indirect effect [40]. Indeed perturbation of several processes results in inhibition of cytokinesis including flagellar attachment, GPI biosynthesis, Golgi duplication and kinetoplast duplication [41,42]. Given such an impact on DNA fidelity, we wanted to investigate the genomic effects of resistance to the oxaborole.

### Generation of oxaborole-resistant cell lines

**Fig 4:**
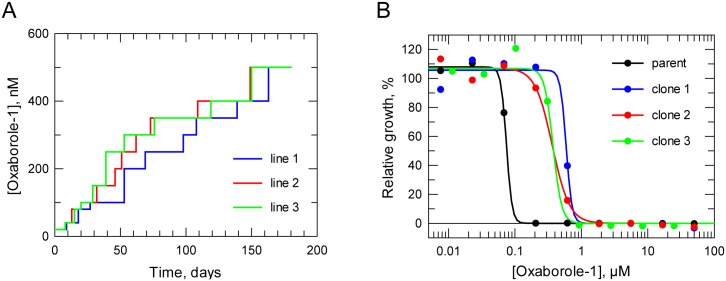
Generation of cell lines resistant to Oxaborole-1. **A.** Stepwise generation of resistance to oxaborole. **B.** Representative drug sensitivity plots. The parental cloned line had an EC_50_ value of 64.4 ± 1.8 nM and the resistant clones 1, 2 and 3 had EC_50_ values of 530 ± 21, 390 ± 17 and 340 ± 27, nM, respectively. Data are the weighted mean of three independent determinations.

In order to investigate the ease with which resistance to the oxaborole could occur, we generated three independent clones of *T*. *brucei* able to sustain growth in 500-nM Oxaborole-1 (Fig 4A). This process took 180 days for all three cell lines to achieve the target of growth in 500 nM Oxaborole-1. A single clone was chosen for each resistant line, and sensitivity to the oxaborole measured by EC_50_ (Fig 4B). The resulting EC_50_ shifts were between 5–8 fold compared to the sensitivity of the parental cell line to Oxaborole-1. A similar process using nifurtimox generated *T*. *brucei* able to grow in >20× EC_50_ after 140 days [11], although after cloning, the shift in sensitivity to nifurtimox was 8-fold. *T*. *brucei* resistant to eflornithine, pentamidine and the methionine tRNA synthetase inhibitor 1433, have all been generated to grow at 32× their EC_50_ concentrations within 120 days or less [43]. Whilst a major motive for investigating mode of action was to aid the protection of the oxaborole class from resistance in the field, these results indicate greater resilience to resistance to the oxaborole class than drugs currently used in the field as well as compounds in development.

### Reversion of oxaborole-resistance

Following two weeks incubation in the absence of Oxaborole-1, an EC_50_ determination showed loss of resistance from cell line 1 (EC_50_ value of 123 ± 12 nM compared to 530 ± 21 nM). This cell line was cloned by limiting dilution and EC_50_ determinations were carried out on five clones. All of the clones showed loss of resistance, the clone with greatest loss of resistance (termed clone 1R) had an EC_50_ value of 83 ± 2 nM (weighted mean of two determinations). Culture of resistant cell lines 2 and 3 failed to show any loss of resistance over eight weeks in the absence of Oxaborole-1.

The apparent greater instability of resistance in resistant cell line 1 was consistent with problems encountered when attempting to revive frozen cells. Stabilated cells revived from resistant cell lines 2 and 3 grew at the same rate as the parental cell line. However, cells from resistant cell line 1 showed little motility, although there were no abnormalities in gross morphology by light microscopy. After 7–14 days growth was regained, however after a single passage and three days of growth to select for healthy cells, resistance had been lost. This suggests at least two routes of resistance, an unstable mechanism and one or more stable mechanisms. Greater stability could be conferred by a gene segment being totally lost rather than silenced, or genomic amplification carrying a significant fitness cost compared to one with no such cost.

### Genomic sequencing

**Fig 5:**
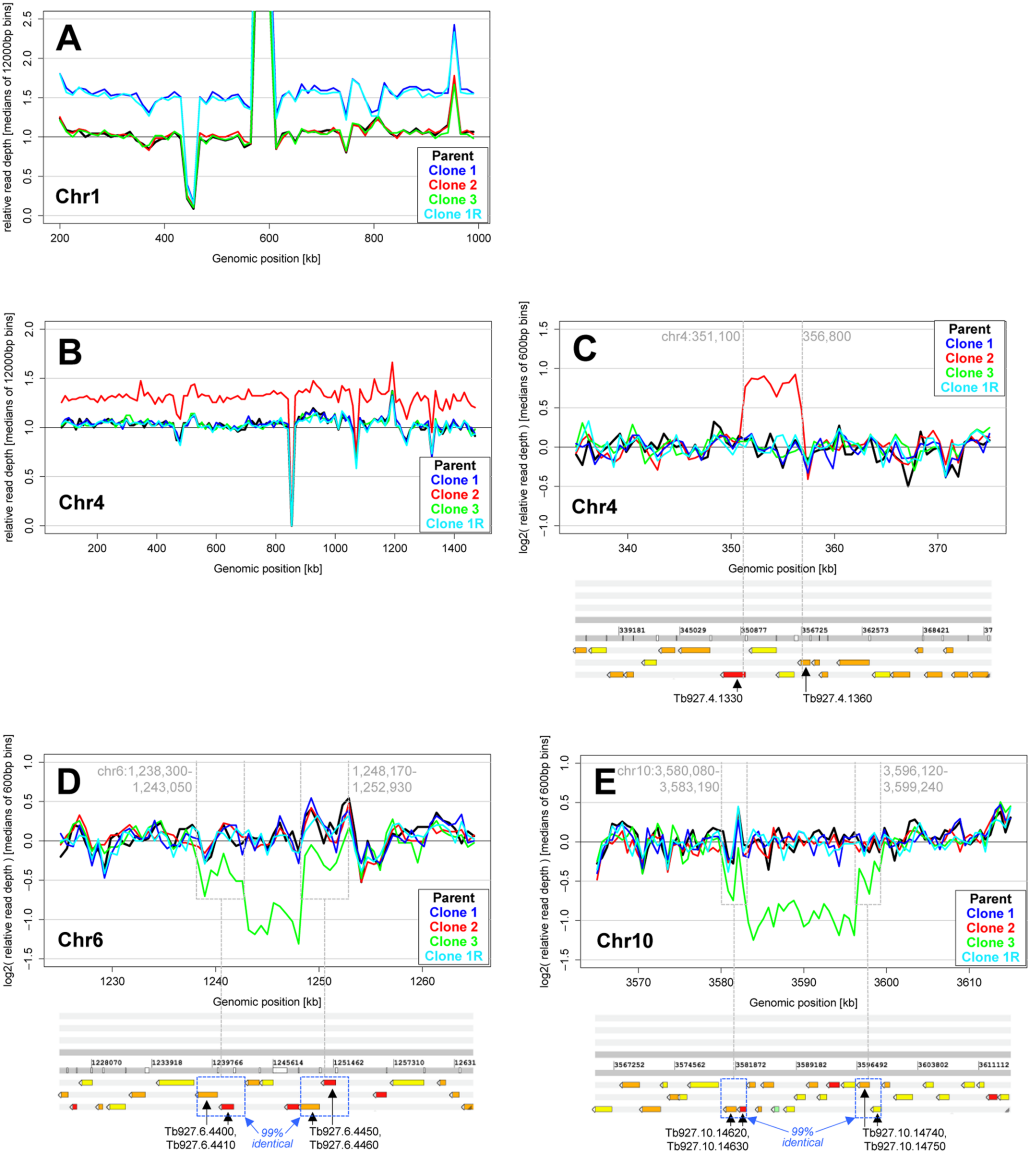
Copy number variations (CNVs) in the drug-resistant and -revertant parasite lines identified by whole genome sequencing. The read coverage plots show the relative read depth across selected chromosomal regions in the different parasite lines. (**A, B**) The panels depict the relative read coverage across the core regions of chromosomes 1 and 4, analysed in windows of 12 kb width, by plotting the ratio of the median read depth observed in a given window over the median read depth of all chromosomes for that parasite line. A relative read depth of 1.5 therefore represents a 50% copy number increase of the respective chromosome compared to the rest of the genome, which indicates an absolute copy number increase from two to three in the diploid parasite genome. (**C-E**) These panels depict the relative read coverage across selected chromosomal regions, analysed in windows of 600 bp width, by plotting the log_2_-based ratio of the median read depth observed in a given window over the median read depth of the entire core region of that chromosome for that parasite line. A log_2_-based relative read depth of 1 therefore represents a 100% copy number increase of the respective region compared to the rest of that chromosome, whereas a log_2_-based relative read depth of -1 indicates a 50% reduction in read depth which is likely due to the loss of one of the two alleles in that region (hemizygosity). In both cases of allele loss, the central area with 50% diminished read depth is flanked by two regions that are approximately 99% identical to one another (D, E). The location of genes along chromosomal regions is indicated below the graphs (C-E). Note the variable but highly reproducible (across the different parasite lines) nature of the read coverage which is in part caused by differences in sequence read mapping efficiency across the genome especially in regions of repetitive sequence.

To identify genetic determinants that may be involved in drug resistance to the oxaborole class, we sequenced the genomes of the susceptible parental strain Lister 427, the three drug-resistant clones and the revertant cell line. We found striking copy number variations (CNVs) between the parasite clones, ranging from apparent whole chromosome duplications to CNVs affecting regions of approximately 5 kb to 15 kb in length (Fig 5). For example, chromosome 1 occurs in three instead of the usual two copies in the genome of clones 1 and 1R (Fig 5A), while chromosome 4 displays an elevated copy number in clone 2 (Fig 5B). In addition, a short region of chromosome 4 of approximately 5.5 kb is further duplicated in this cell line (Fig 5C), thereby providing further complete copies of the two genes *CPSF3* (a putative cleavage and polyadenylation specificity factor subunit, Tb927.4.1340) and *glx2-2* (a glyoxalase, Tb927.4.1350) (Table 2). In contrast, two short regions on chromosome 6 and chromosome 10 in drug-resistant clone 3 have lost one of their two alleles (Fig 5D and 5E). Interestingly, the deleted regions are flanked in both cases by shorter regions with nearly 100% sequence identity: on chromosome 6 the central, deleted region of 5.1 kb is flanked by two near-identical regions of approximately 4.8 kb each, whereas on chromosome 10 it is a central region of 12.9 kb that is flanked by two near-identical regions of approximately 3.1 kb each (Fig 5D and 5E). This suggests homologous crossover as the mechanism of DNA deletion in these cases. These deletions directly affect over a dozen genes (Table 2) and render the affected regions hemizygous, an observation that is confirmed by the loss of a second allele in the genotype of some of these genes (S2 Table).

**Table 2:** Genes affected by copy number variations (CNVs) observed in the parasite lines.

Chromosome	Gene ID	Gene Description	Change	Parasite Line
4	Tb927.4.1330	DNA topoisomerase IB, large subunit	partial amplification	2
4	Tb927.4.1340	cleavage and polyadenylation specificity factor subunit, putative (CPSF3)	amplification	2
4	Tb927.4.1350	glyoxalase II, hydroxyacylglutathione hydrolase, putative (glx2-2)	amplification	2
4	Tb927.4.1360	hypothetical protein, conserved	partial amplification	2
6	Tb927.6.4400 #	hypothetical protein, conserved	deletion	3
6	Tb927.6.4410 #	S-adenosylmethionine decarboxylase (AdoMetDC)	deletion	3
6	Tb927.6.4420	hypothetical protein, conserved	deletion	3
6	Tb927.6.4430	homoserine kinase, putative (HK)	deletion	3
6	Tb927.6.4440	RNA-binding protein 42 (RNA-binding motif protein 42) (RBP42)	deletion	3
6	Tb927.6.4450 ##	hypothetical protein, conserved	deletion	3
6	Tb927.6.4460 ##	S-adenosylmethionine decarboxylase (AdoMetDC)	deletion	3
10	Tb927.10.14620 *	hypothetical protein, conserved	deletion	3
10	Tb927.10.14630 *	fibrillarin, putative	deletion	3
10	Tb927.10.14640	hypothetical protein, conserved	deletion	3
10	Tb927.10.14650	hypothetical protein, conserved	deletion	3
10	Tb927.10.14660	hypothetical protein, conserved	deletion	3
10	Tb927.10.14670	hypothetical protein	deletion	3
10	Tb927.10.14680	ribosome biogenesis protein, putative	deletion	3
10	Tb927.10.14690	syntaxin, putative	deletion	3
10	Tb927.10.14700	hypothetical protein, conserved	deletion	3
10	Tb927.10.14710	40S ribosomal protein S2, putative (RPS2)	deletion	3
10	Tb927.10.14720	peroxin 13,SH3 domain protein, conserved (PEX13)	deletion	3
10	Tb927.10.14730	chaperone protein DNAj, putative	deletion	3
10	Tb927.10.14740 **	hypothetical protein, conserved	deletion	3
10	Tb927.10.14750 **	fibrillarin, putative	deletion	3

Note: Genes affected by CNVs of whole chromosomes (chromosomes 1 and 4 in clone 1/clone 1 revertant and clone 2, respectively) are not listed.

The genes marked with single and double hash symbols or single and double asterisks are part of regions that are approximately 99% identical to one another, e.g. a 4.8kb-long region including Tb927.6.4400 and Tb927.6.4410 is 99% identical to a region including Tb927.6.4450 and Tb927.6.4460.

The comparison of genotype assignments between the drug-resistant lines and those of the parental strain uncovered in total 78 single nucleotide polymorphisms (SNPs) in 66 genes. Of these, 41 in 38 genes are predicted to result in non-synonymous amino acid changes that could potentially contribute to the observed drug resistance phenotypes (S2 Table). Only one SNP was common to all four clones (receptor-type adenylate cyclase GRESAG 4, putative), but no SNP was common to all 3 resistant clones and absent from the revertant clone, as might be expected if a single point mutation in a single gene was responsible for resistance. Likewise, the small ubiquitin-related modifier (SUMO) contained different SNPs in all 4 clones. SUMOylation regulates a wide variety of cellular processes, including transcription, mitotic chromosome segregation, DNA replication and repair and ribosomal biogenesis [44,45] and offers an attractive explanation for the chromosomal abnormalities described above. Knock down of SUMO in procyclic forms of *T*. *brucei* results in arrest in G_2_/M phase of the cell cycle as observed here [46]. In the case of clone 3, the SNP results in replacement of the initiator methionine residue with an isoleucine. Inspection of the flanking region of SUMO revealed no upstream in-frame methionine and the next downstream methionine is at residue 50 in this 114 amino acid protein. Based on the solution structure of *T*. *brucei* SUMO [47], the truncated protein is likely to be non-functional. In the other two clones, the SNPs are located at the C-terminus of the 114 residue peptide close to (Ala101Gly, clone 2) or adjacent to (Thr106Ile, clone 1) the site of cleavage by ULP1/SENP which reveals a C-terminal di-glycine motif required for activation of SUMO by the E1 activating complex [45]. Ala101 maps to a region predicted to interact with the SUMO conjugating enzyme E2 (Ubc9) [47], but it is difficult to predict whether or not such a conservative substitution with a glycine would significantly alter the interaction of the enzyme with its substrate. Cells expressing Thr106Arg or Thr106Lys SUMO mutants have been used in a proteomic study to successfully identify SUMO targets in *T*. *brucei* [48] so it appears possible that an isoleucine would also be tolerated at this position. Furthermore, this SNP is retained in the revertant clone, suggesting this mutation is not involved in resistance. Nevertheless, it is possible that different resistance mechanisms may have arisen in each of these lines. None of the genes potentially involved in SUMOylation [49] were found in common with either our proteomic or our genomic studies. However, of the 44 proteins identified as SUMOylated in a previous study [48], one gene Tb927.4.1330 (DNA topoisomerase 1B, large subunit) was identified as duplicated in resistant clone 2 (Table 2). This 90 kDa protein has 4 SUMOylation sites [48] and forms a functional heterodimer with a 36 kDa catalytic subunit and is essential for growth of the parasite [50]. However, it is noteworthy that topoisomerase-IIα, a SUMOylated protein in other organisms, is essential in bloodstream form *T*. *brucei* for centromere-specific topoisomerase cleavage activity [48], but was not present in any of our candidate lists

Another candidate in the genome sequencing data, *T*. *brucei* homoserine kinase, has recently been studied in our laboratory in relation to *de novo* synthesis of threonine [51]. The recombinant enzyme was completely insensitive to inhibition by Oxaborole-1 (up to 50 μM) and therefore is not the target for this compound. To investigate if the deletion of a copy homoserine kinase (CNV in resistant line 3, Table 2) was implicated in resistance, the sensitivity to Oxaborole-1 in wild-type (WT), single knockout (SKO^PAC^) and double knockout (DKO) bloodstream forms was determined. The resulting EC_50_ values were all within experimental error of each other (41.1 ± 1.6, 36.1 ± 1.3 and 37.5 ± 1.5 nM for WT, SKO^PAC^ and DKO, respectively). Taken together, we can conclude that HSK is neither the target nor a resistance determinant for oxaborole compounds.

This list of candidate mode of action genes, affected either by CNVs or the presence of SNPs, is too long to be systematically investigated. Since no genes are common to both the proteomic studies and the resistant studies, genes involved in resistance mechanisms would appear to be distinct from candidate proteins implicated in the mode of action. In addition, the genomic variations we have observed could be the result of either oxaborole exposure or an unidentified resistance mechanism resulting in a general loss of DNA fidelity.

In conclusion, genetic analysis of laboratory-generated resistant lines has been an effective technique when the field can be narrowed to particular genes of interest as in the case of resistance of *T*. *brucei* to tRNA synthetase inhibitors resulting from overexpression of the target [43]. However, taking an unbiased whole genome sequencing approach alongside the analysis of oxaborole-binding proteins in the current study, has revealed too many candidates to embark on a systematic appraisal.

Our SILAC-based analysis suggests considerable polypharmacology consistent with the unusually long time taken to develop resistance, apparent multiple routes to resistance and lack of stability in at least one of those routes. It should be borne in mind that resistance and/or mode of action may involve several candidates acting in concert.

The surprising number of large-scale genomic aberrations in our resistant cell lines (Fig 5), and the accumulation of cells in G2/>G2 (Fig 3) suggest DNA fidelity as an area of specific interest. The presence of SNPs in the gene for SUMO (S2 Table) is particularly striking as its repertoire of targets includes proteins involved in chromatin structure and DNA repair [52]. Future work will involve selecting candidates to test by protein modulation and sensitivity to oxaboroles in the whole cell.

## Supporting Information

S1 Text Chemical syntheses. (DOCX)

S1 Fig Synthesis of SCYX-6759, Oxaborole-1 and Oxaborole-2. (PPTX)

S2 Fig The synthesis of Oxaborole-3 and Oxaborole-Resin. (PPTX)

S3 Fig Synthesis of Control-1, Control-Biotin and Control-Resin. (PPTX)

S1 Table Quantitative proteomic analysis of Oxaborole targets. (XLSX)

S2 Table Genomic variants predicted to cause non-synonymous amino acid changes in drug-resistant and -revertant parasites in comparison to parental strain Lister 427. (XLSX)

S1 Dataset Variant Call Format file for WT oxaborole-susceptible line. (GZ)

S2 Dataset Variant Call Format file for oxaborole-resistant clone 1. (GZ)

S3 Dataset Variant Call Format file for oxaborole-resistant clone 2. (GZ)

S4 Dataset Variant Call Format file for oxaborole-resistant clone 3. (GZ)

S5 Dataset Variant Call Format file for oxaborole-revertant clone 1R. (GZ)
