# Supplementary material for: Genomic and Proteomic Studies on the Mode of Action of Oxaboroles against the African Trypanosome
Source: PLoS Negl Trop Dis. 2015 Dec 18;9(12):e0004299. doi: 10.1371/journal.pntd.0004299 (PMC4689576; doi:10.1371/journal.pntd.0004299)
Supplement: S1 Text — (DOCX) [file pntd.0004299.s001.docx]

**S1 Chemical Syntheses**

**Chemistry general**

Chemicals and solvents were purchased from Sigma-Aldrich, Alfa Aesar, Apollo Scientific, Fisher Chemicals, TCI UK and VWR, and were used as received. Air and moisture sensitive reactions were carried out under an inert atmosphere of nitrogen. Analytical thin-layer chromatography (TLC) was performed using pre-coated TLC plates (layer 0.20 mm silica gel 60 with fluorescent indicator UV254, from Merck). Developed plates were air-dried and analyzed under a UV lamp (UV254/365 nm), and/or with chemical stains where appropriate. Flash column chromatography was performed using prepacked silica gel cartridges (230-400 mesh, 35-70 μm, from Teledyne ISCO) using a Teledyne ISCO CombiFlash Rf, or a Teledyne ISCO CombiFlash Companion. ^1^H-NMR, ^13^C-NMR, ^19^F-NMR, and 2D-NMR spectra were recorded on a Bruker Avance DPX 500 spectrometer (^1^H at 500.1 MHz, ^13^C at 125.8 MHz, ^19^F at 470.5 MHz), or a Bruker Avance III HD (^1^H at 400.1 MHz, ^13^C at 100.6 MHz). Chemical shifts (δ) are expressed in ppm recorded using the residual solvent as the internal reference in all cases. Signal splitting patterns are described as singlet (s), doublet (d), triplet (t), quartet (q), multiplet (m), broad (br), or a combination thereof. Coupling constants (*J*) are quoted to the nearest 0.5 Hz. LC-MS analyses were performed with either an Agilent HPLC 1100 series connected to a Bruker Daltonics MicrOTOF or an Agilent Technologies 1200 series HPLC connected to an Agilent Technologies 6130 quadrupole LC/MS, where both instruments were connected to an Agilent diode array detector. LCMS chromatographic separations were conducted with either a Waters XBridge C18 column, 50 mm × 2.1 mm, 3.5 μm particle size, or Waters XSelect C18 column, 30 mm × 2.1 mm, 2.5 μm particle size; mobile phase, water/acetonitrile +0.1% HCOOH, or water/acetonitrile +0.1% NH_3_. High-resolution electrospray measurements were performed on a Bruker Daltonics MicrOTOF mass spectrometer. Preparative HPLC separations were performed with a Gilson HPLC (321 pumps, 819 injection module, 215 liquid handler/injector) connected to a Gilson 155 UV/vis detector, or a Waters mass-directed HPLC (system fluidics organizer, 2545 binary gradient module, 2×515 HPLC pumps, 2767 sample manager) connected in parallel to a Waters 3100 mass detector and a 2998 photodiode array detector. HPLC chromatographic separations were conducted using a Waters XBridge C18 column, 19×100 mm, 5 μm particle size; mobile phase, water/acetonitrile +0.1% NH_3_, or HCOOH.

**Chemical syntheses**

Known bioactive oxaboroles **SCYX-6759** and **Oxaborole-1** were prepared using a modification of published synthetic procedures [1] from commercially available aniline **I** (Figure S1). Reaction between the activated biotin ester **II** and the same aniline **I** in the presence of base furnished **Oxaborole-2** in low yield (Figure S1).

The synthesis of an oxaborole derivative suitable for immobilisation onto a resin either directly, or via conjugation with biotin is shown in Figure S2. Briefly, the known amino alcohol **III** [2] was converted to the tosylate **IV**, which was in turn used to prepare a phenolic ether containing an ester functionality (**V**) by reaction with methyl 3-hydroxybenzoate and base. Ester **V** was subsequently hydrolysed under basic conditions to give acid **VI**, which was reacted with the oxaborole aniline **I** to give an oxaborole analogue containing a PEG linker (**Oxaborole-3**). The Boc group of **Oxaborole-3** was removed under acidic conditions to give the primary amine **VII**, which was either reacted with an activated ester of biotin (**II**), or NHS-functionalised beads to give the **Oxaborole-Biotin**, or **Oxaborole-Resin** respectively.

The phthalide bicycle was used as a replacement of the oxaborole ring system in order to provide the structurally-related inactive negative control compounds **Control-1**, **Control-Biotin** and **Control-Resin** (Figure S3). The chemical routes utilised in the control syntheses were closely related to the synthesis employed to prepare the active oxaborole-controlling analogues (see Figures S1 and S2)

**Synthesis of Oxaborole-1 (*N*-(1-hydroxy-1,3-dihydrobenzo[*c*][1,2]oxaborol-6-yl)benzenesulfonamide).**

This was prepared using a modification of that described in Ding *et al* [1]. Briefly, DIPEA (129 mg, 1 mmol) was added to a suspension of 6-aminobenzo[*c*][1,2]oxaborol-1(3*H*)-ol hydrochloride (**I**, Apollo Scientific) (93 mg, 0.5 mmol) in anhydrous THF (2.5 mL) at 0°C and stirred for 1 h. The reaction was subsequently diluted with anhydrous MeCN (2.5 mL) followed by the addition of benzenesulfonyl chloride (102 mg, 0.575 mmol). The reaction was then allowed to warm to room temperature and stirred for an additional 16 h. Workup was initiated by the addition of AcOH (0.5 mL), after which the reaction was stirred for 1 h and the solvents removed under reduced pressure. The resultant crude residue was purified by reverse phase preparative HPLC (5:95→95:5 MeCN:water+0.1% HCOOH) to give the title compound as an off-white solid (97 mg, 67% yield). ^1^H-NMR (500 MHz, DMSO-*d*_6_) δ 10.26 (1H, br s, NH), 9.24 (1H, s, OH), 7.75-7.73 (2H, m, 2×ArH), 7.62-7.58 (1H, m, ArH), 7.56-7.52 (2H, m, 2×ArH), 7.49 (1H, d, *J*=2.0 Hz, ArH), 7.26 (1H, dd, *J*=8.0, 0.5 Hz, ArH), 7.18 (dd, *J*= 8.0, 2.0 Hz, ArH), 4.88 (2H, s, CH_2_). ^13^C-NMR (125 MHz, DMSO-*d*_6_) δ 149.8 (C), 139.5 (C), 136.5 (C), 132.8 (CH), 129.2 (CH), 126.6 (CH), 123.7 (CH), 122.4 (CH), 122.0 (CH), 69.5 (CH_2_). [note, one quaternary carbon resonance is not observed]. MS (ES-): *m/z* (%) 288 (96) [M-H]^-^, 559 (100) [2M-H_2_O-H]^-^. HRMS (ES-): calcd for C_13_H_11_B_1_N_1_O_4_S_1_ [M-H]^-^ 288.0507, found 288.0494 (4.7 ppm).

**Synthesis of SCYX-6759 (4-fluoro-*N*-(1-hydroxy-1,3-dihydrobenzo[*c*][1,2]
oxaborol-6-yl)-2-(trifluoromethyl)benzamide).**

Neat DIPEA (129 mg, 1 mmol) was added to a stirred suspension of aniline **I** (93 mg, 0.5 mmol) in anhydrous THF (5 mL) at 0°C. After 1 h, neat acid 4-fluoro-2-(trifluoro
methyl)benzoyl chloride (130 mg, 0.58 mmol) was added and the reaction mixture allowed to warm to room temperature, and stirred for an additional 16 h. Workup was initiated by the addition of MeOH/AcOH (1:1, 2 mL), after 20 min stirring the reaction solvent was removed under reduced pressure and the crude residue subjected to silica column chromatography (12 g silica, 0:100→20:80 0.5% AcOH in MeOH:CH_2_Cl_2_). The resultant product was further purified by reverse phase HPLC (5:95-95:5 MeCN:water+0.1% HCOOH) to give the title compound as a white solid (75 mg, 44%). ^1^H-NMR (500 MHz, DMSO-*d*_6_) δ 10.62 (s, 1H, NH), 9.29 (s, 1H, OH), 8.15 (d, 1H, *J*=2.0 Hz, ArH), 7.84-7.81 (m, 1H, 2×ArH), 7.72-7.68 (m, 1H, ArH), 7.66 (dd, 1H, *J*=8.0, 2.0 Hz, ArH), 7.40 (d, 1H, *J*=8.0 Hz, ArH), 4.97 (s, 2H, CH_2_). ^19^F-NMR (470 MHz, DMSO-*d*_6_) δ -58.4 (CF_3_), -109.3 (ArF). ^13^C-NMR (125 MHz, DMSO-*d*_6_) δ 164.6 (C), 161.9 (d, *J*=247 Hz, CF), 149.5 (C), 137.6 (C), 133.0 (C), 131.4 (d, *J*=8.0 Hz, CH), 122.8 (CH), 121.6 (2×CH), 119.5 (d, *J*=21.0 Hz, CH), 114.3-114.0 (m, CH). Note, 3 carbon resonances are absent. HMBC suggests that one quaternary carbon resonance is coincident with the CH resonance at 131.4 ppm. Other peaks are likely to be of low intensity due to C-F coupling. MS (ES+): *m/z* (%) 340 (100) [M+H]^+^, 398 (27) [M+NH_4_+MeCN]^+^. HRMS (ES-): calcd for C_15_H_9_B_1_F_4_N_1_O_3_ [M-H]^-^ 338.0617, found 338.0614 (1.1 ppm).

**Synthesis of biotin pentafluorophenyl ester (II).**

Neat DIC (757 mg, 6 mmol) was added to a solution of biotin (977 mg, 4 mmol) and pentafluorophenol (957 mg, 5.2 mmol) in anhydrous DMAc (15 mL) at 0°C. The reaction was subsequently allowed to warm to room temperature and stirred for an additional 64 h. The solvent was then removed under reduced pressure and the resultant crude product recrystallised from hot MeOH to give the title compound as a white solid (1.10 g, 67%). ^1^H-NMR (500 MHz, DMSO-*d*_6_) δ 6.47 (br s, 1H, NH), 6.38 (br s, 1H, NH), 4.32-4.30 (m, 1H, CH), 4.16-4.13 (m, 1H, CH), 3.14-3.10 (m, 1H, CH), 2.85-2.78 (m, 3H, CH_2_ & C*H*H), 2.59-2.57 (m, 1H, C*H*H), 1.73-1.62 (m, 3H, CH_2_ & C*H*H), 1.55-1.38 (m, 3H, CH_2_ & C*H*H). ^19^F-NMR (470 MHz, DMSO-*d*_6_) δ -153.5 - -153.6 (m, 2F, 2×ArF), -158.0 - -158.1 (m, 1H, ArF), -162.5 - -162.6 (m, 2H, 2×ArF). MS (ES+): *m/z* (%) 821 (100) [2M+H]^+^.

**Synthesis of oxaborole-2 (*N*-(1-hydroxy-1,3-dihydrobenzo[*c*][1,2]oxaborol-6-yl)-5-((3a*S*,4*S*,6a*R*)-2-oxohexahydro-1*H*-thieno[3,4-*d*]imidazol-4-yl)pentanamide).**

Neat DIPEA (129 mg, 1 mmol) was added to a stirred suspension of aniline **I** (93 mg, 0.5 mmol) in anhydrous THF (5 mL) at 0°C. After 15 min, a suspension of biotin ester **II** (236 mg, 0.575 mmol) in THF (5 mL) was added and the reaction mixture allowed to warm to room temperature, and stirred for an additional 16 h. The reaction solvent was then removed under reduced pressure, the residue triturated with MeCN and the resultant solid recovered by filtration. The crude product was purified by reverse phase HPLC (5:95→95:5 MeCN:water+0.1% HCOOH) to give the title compound as a yellow solid (6 mg, 3%). ^1^H-NMR (500 MHz, DMSO-*d*_6_) δ 9.91 (s, 1H, NH), 9.21 (s, 1H, OH), 8.01 (d, 1H, *J*=2.0 Hz, ArH), 7.61 (dd, 1H, *J*=8.5, 2.0 Hz, ArH), 7.32 (d, 1H, *J*=8.5 Hz, ArH), 6.47 (br s, 1H, NH), 6.39 (br s, 1H, NH), 4.94 (s, 2H, CH_2_), 4.33-4.30 (m, 1H, CH), 4.16-4.14 (m, 1H, CH), 3.15-3.12 (m, 1H, CH), 2.83 (dd, 1H, *J*=12.5, 5.0 Hz, C*H*H), 2.59 (d, 1H, *J*=12.5 Hz, C*H*H), 2.34-2.31 (m, 2H, CH_2_), 1.70-1.58 (m, 3H, CH_2_ & C*H*H), 1.55-1.48 (m, 1H, C*H*H), 1.45-1.34 (m, 2H, CH_2_). ^13^C-NMR (125 MHz, DMSO-*d*_6_) δ 171.1 (C), 162.7 (C), 148.4 (C), 138.1 (C), 122.2 (CH), 121.4 (CH), 120.9 (CH), 69.6 (CH_2_), 61.0 (CH), 59.2 (CH), 55.4 (CH), 39.9 (CH_2_), 36.1 (CH_2_), 28.2 (CH_2_), 28.1 (CH_2_), 25.2 (CH_2_). Note, one quaternary carbon resonance was not detected. MS (ES+): *m/z* (%) 376 (100) [M+H]^+^, 733 (65) [2M-H_2_O]^+^. HRMS (ES+): calcd for C_17_H_22_B_1_N_3_Na_1_O_4_S_1_ [M+Na]^+^ 398.1316, found 398.1322 (-1.4 ppm).

**Synthesis of intermediate IV (2-(2-((*tert*-butoxycarbonyl)amino)ethoxy)ethyl 4-methylbenzenesulfonate).**

Neat DIPEA (2.58 g, 20 mmol) was added to a solution of **III** [2] (2.05 g, 10 mmol), TsCl (3.81 g, 20 mmol) and DMAP (12 mg, 0.1 mmol) in anhydrous CH_2_Cl_2_ (50 mL) at 0°C. The reaction was then allowed to warm to room temperature and stirred for an additional 16 h, after which the reaction was washed with satd. aq. NH_4_Cl (50 mL) and the layers separated. The aq. layer was subsequently extracted with CH_2_Cl_2_ (3×50 mL), the CH_2_Cl_2_ layers combined, dried over MgSO_4_, filtered and the solvent removed under reduced pressure. The crude product was purified by chromatography (120 g silica, 0:100→100:0 EtOAc:hexane) to give the title compound as a clear oil (1.02 g, 28%). Rf (silica, 50:50 EtOAc:hexane) 0.61. ^1^H-NMR (500 MHz, CDCl_3_) δ 7.84-7.80 (m, 2H, AA′BB′, 2×ArH), 7.38-7.35 (m, 2H, AA′BB′, 2×ArH), 4.81 (br s, 1H, NH), 4.19-4.16 (m, 2H, CH_2_), 3.65-3.62 (m, 2H, CH_2_), 3.47-3.44 (m, 2H, CH_2_), 3.27-3.22 (m, 2H, CH_2_), 2.46 (s, 3H, CH_3_), 1.46 (s, 9H, 3×CH_3_). MS (ES+): *m/z* (%) 260 (100) [M-Boc+H]^+^, 382 (15) [M+Na]^+^.

**Synthesis of intermediate V (methyl 3-(2-(2-((*tert*-butoxycarbonyl)amino)ethoxy)
ethoxy)benzoate).**

A solution of tosylate **IV** (863 mg, 2.4 mmol) in anhydrous MeCN (5 mL) was added to a suspension of methyl 3-hydroxybenzote (304 mg, 2 mmol) and K_2_CO_3_ (332 mg, 2.4 mmol) in anhydrous MeCN (5 mL) and stirred at 82°C for 16 h. The reaction was subsequently filtered, the recovered solid washed with MeCN (3×5 mL), and the combined organics removed under reduced pressure. The crude product was purified by chromatography (40 g silica, 10:90→60:40 EtOAc:hexane) to give the title compound as a clear oil (651 mg, 96%). Rf (silica, 20:80 EtOAc:hexane) 0.19. ^1^H-NMR (500 MHz, CDCl_3_) δ 7.66-7.64 (m, 1H, ArH), 7.60 (dd, 1H, *J*=2.5, 1.0 Hz, ArH), 7.35 (dd, 1H, *J*=8.0, 8.0 Hz, ArH), 7.15 (ddd, 1H, *J*=8.0, 2.5, 1.0 Hz, ArH), 4.99 (br s, 1H, NH), 4.19-4.17 (m, 1H, CH_2_), 3.92 (s, 3H, CH_3_), 3.85-3.83 (m, 2H, CH_2_), 3.63-3.61 (m, 2H, CH_2_), 3.37-3.34 (m, 2H, CH_2_), 1.44 (s, 3H, CH_3_). ^13^C-NMR (100 MHz, CDCl_3_) δ 166.8 (C), 158.7 (C), 156.0 (C), 131.4 (C), 129.4 (CH), 122.2 (CH), 120.1 (CH), 114.7 (CH), 79.1 (C), 70.4 (CH_2_), 69.4 (CH_2_), 67.5 (CH_2_), 52.1 (CH_3_), 40.3 (CH_2_), 28.4 (CH_3_). MS (ES+): *m/z* (%) 240 (100) [M-Boc+H]^+^, 362 (60) [M+Na]^+^.

**Synthesis of Intermediate VI (3-(2-(2-((*tert*-butoxycarbonyl)amino)ethoxy)
ethoxy)benzoic acid).**

A solution of 2M aq NaOH (6.35 mL, 12.7 mmol) was added to a solution of ester **V** (430 mg, 1.27 mmol) in THF (5 mL) and stirred at room temperature for 16 h, followed by heating at 70°C for 2 h. The reaction mixture was subsequently diluted with EtOAc (20 mL), the layers separated and the pH of the aqueous adjusted to 3. The aq was then extracted with EtOAc (3×20 mL), the organic layers combined, dried over MgSO_4_, filtered and the solvent removed under reduced pressure to give the title compound as a white solid (349 mg, 84%) which was used without further purification. ^1^H-NMR (500 MHz, CDCl_3_) δ 7.71 (d, 1H, *J*=8.0 Hz, ArH), 7.64 (br s, 1H, ArH), 7.38 (dd, 1H, *J*=8.0, 8.0 Hz, ArH), 7.19 (dd, 1H, *J*=8.0, 2.0 Hz, ArH), 5.03 (br s, 1H, NH), 4.20-4.18 (m, 2H, CH_2_), 3.86-3.84 (m, 2H, CH_2_), 3.64-3.62 (m, 2H, CH_2_), 3.38-3.35 (m, 2H, CH_2_), 1.45 (s, 9H, 3×CH_3_). MS (ES+): *m/z* (%) 326 (25) [M+H]^+^, 668 (100) [2M+NH_4_]^+^.

**Synthesis of Oxaborole-3 (*tert*-butyl (2-(2-(3-((1-hydroxy-1,3-dihydrobenzo[*c*]
[1,2]oxaborol-6-yl)carbamoyl)phenoxy)ethoxy)ethyl)carbamate).**

Step 1: neat DIPEA (78 mg, 0.604 mmol) was added to a solution of acid **VI** (187 mg, 0.575 mmol) and TBTU (231 mg, 0.719 mmol) in anhydrous THF (10 mL) at 0°C. The reaction was allowed to warm to room temperature and stirred for an additional 20 h. Step 2: neat DIPEA (129 mg, 1.0 mmol) was added to a suspension of aniline **I** (93 mg, 0.5 mmol) in anhydrous THF (5 mL) at 0°C and stirred for 2 h, before being diluted with MeCN (2.5 mL). Step 3: the activated ester from step 1 was cooled to 0°C and added to the aniline free base from step 2. The resultant reaction was allowed to warm to room temperature and stirred for an additional 16 h. The reaction solvent was then removed under reduced pressure and the resultant crude dissolved in DMF, filtered and purified directly by reverse phase HPLC (5:95-40:60 MeCN:water+0.1% NH_3_) to give the title compound as a yellow solid (119 mg, 52%).

^1^H-NMR (400 MHz, DMSO-*d*_6_) δ 10.27 (s, 1H, NH), 9.26 (s, 1H, OH), 8.17 (d, 1H, *J*=2.0 Hz, ArH), 7.77 (dd, 1H, *J*=7.0, 2.0 Hz, ArH), 7.56-7.53 (m, 2H, 2×ArH), 7.45 (dd, 1H, *J*=8.0 Hz, ArH), 7.39 (d, 1H, *J*=8.0 Hz, ArH), 7.18 (ddd, 1H, *J*=8.0, 2.5, 1.0 Hz, ArH), 6.85 (br t, 1H, *J*=6.0 Hz, NH), 4.98 (s, 2H, CH_2_), 4.19-4.18 (m, 2H, CH_2_), 3.78-3.76 (m, 2H, CH_2_), 3.48-3.46 (m, 2H, CH_2_), 3.13-3.09 (m, 2H, CH_2_), 1.37 (s, 9H, 3×CH_3_). ^13^C-NMR (100 MHz, DMSO-*d*_6_) δ 165.6 (C), 158.8 (C), 156.1 (C), 149.7 (C), 138.3 (C), 136.8 (C), 131.3 (C), 130.0 (CH), 124.2 (CH), 123.0 (CH), 121.9 (CH), 120.5 (CH), 118.1 (CH), 114.0 (CH), 78.1 (C), 70.2 (CH_2_), 69.8 (CH_2_), 69.1 (CH_2_), 67.8 (CH_2_), 40.1 (CH_2_), 28.7 (CH_3_). MS (ES+): *m/z* (%) 357 (100) [M-Boc+H]^+^, 457 (13) [M+H]^+^. HRMS (ES+): calcd for C_23_H_29_B_1_N_2_Na_1_O_7_ [M+Na]^+^ 479.1960, found 479.1937 (3.5 ppm).

**Synthesis of VII (3-(2-(2-aminoethoxy)ethoxy)-*N*-(1-hydroxy-1,3-dihydrobenzo
[*c*][1,2]oxaborol-6-yl)benzamide).**

TFA (0.25 mL) was added to a solution of **Oxaborole-3** in anhydrous CH_2_Cl_2_ (4.5 mL) at 0°C and stirred for 2 h. The reaction solvent was subsequently removed under reduced pressure, and the resultant crude material purified by preparative HLPC (20:80-95:5 MeCN:water+0.1% HCOOH) to give the title compound as a white solid (30 mg, 96%). ^1^H-NMR (500 MHz, CD_3_OD) δ 7.98 (d, 1H, *J*=2.0 Hz, ArH), 7.81 (dd, 1H, *J*=8.0, 2.0 Hz, ArH), 7.58-7.55 (m, 2H, 2×ArH), 7.46 (dd, 1H, *J*=8.0, 8.0 Hz, ArH), 7.43-7.41 (m, 1H, ArH), 7.19 (ddd, 1H, *J*=8.0, 2.5, 1.0, ArH), 5.10 (s, 2H, CH_2_), 4.30-4.28 (m, 2H, CH_2_), 3.96-3.94 (m, 2H, CH_2_), 3.83-3.81 (m, 2H, CH_2_), 3.20-3.17 (m, 2H, CH_2_). MS (ES+): *m/z* (%) 357 (100) [M+H]^+^.

**Synthesis of Oxaborole-Biotin (*N*-(1-hydroxy-1,3-dihydrobenzo[*c*][1,2]oxaborol-6-yl)-3-(2-(2-(5-((3a*S*,4*S*,6a*R*)-2-oxohexahydro-1*H*-thieno[3,4-*d*]imidazol-4-yl)
pentanamido)ethoxy)ethoxy)benzamide).**

A solution of HCl (4.0M in dioxane, 1 mmol, 1.25 mL) was added to a suspension of **Oxaborole-3** (0.05 mmol, 23 mg) in anhydrous THF (0.75 mL) at 0°C. The reaction was then allowed to warm to room temperature and stirred for an additional 72 h, after which the solvent was removed under reduced pressure. A solution of **II** (0.0575 mmol, 24 mg) in anhydrous THF (5 mL) was then added to the crude primary amine, followed by the addition of neat DIPEA (0.5 mmol, 65 mg). The resultant reaction mixture was stirred at room temperature for 48 h, before the reaction solvent was removed and the crude material purified directly by preparative HPLC (5:95→95:5 MeCN:water+0.1% NH_3_) to give the title compound as a white solid (14 mg, 48%). ^1^H-NMR (500 MHz, DMSO-d_6_) δ 10.28 (s, 1H, NH), 9.27 (s, 1H, OH), 8.18 (d, 1H, *J*=1.5 Hz, ArH), 7.90 (t, 1H, *J*=6.0 Hz, NH), 7.77 (dd, 1H, *J*=8.5, 1.5 Hz, ArH), 7.57-7.54 (m, 2H, 2×ArH), 7.45 (dd, 1H, *J*=8.0 Hz, ArH), 7.40 (d, 1H, *J*=8.5 Hz, ArH), 7.18 (ddd, 1H, *J*=8.0, 2.5, 1.0 Hz, ArH), 6.44 (s, 1H, NH), 6.38 (s, 1H, NH), 4.98 (s, 2H, CH_2_), 4.30-4.27 (m, 1H, CH), 4.20-4.18 (m, 2H, CH_2_), 4.12-4.10 (m, 1H, CH), 3.79-3.77 (m, 2H, CH_2_), 3.51-3.48 (m, 2H, CH_2_), 3.25-3.22 (m, 2H, CH_2_), 3.09-3.06 (m, 1H, CH), 2.82-2.78 (m, 1H, C*H*H), 2.58-2.56 (m, 1H, C*H*H), 2.07 (t, 2H, *J*=7.0 Hz, CH_2_), 1.63-1.56 (m, 1H, C*H*H), 1.55-1.41 (m, 3H, CH_2_&C*H*H), 1.36-1.22 (m, 2H, CH_2_). MS (ES+): *m/z* (%) 583 (100) [M+H]^+^. HRMS (ES+): calcd for C_28_H_35_B_1_N_4_Na_1_O_7_S_1_ [M+Na]^+^ 605.2212, found 605.2182 (5.0 ppm).

**Synthesis of Control-1 (4-fluoro-N-(3-oxo-1,3-dihydroisobenzofuran-5-yl)-2-(trifluoromethyl)benzamide).**

**Control-1** was prepared using a modification of the general procedure described in Jacobs *et al* [3]. Briefly, neat DIPEA (98 mg, 0.97 mmol) was added to a suspension of 4-fluoro-2-(trifluoromethyl)benzoyl chloride (87 mg, 0.39 mmol) and aniline **VIII** (48 mg, 0.32 mmol) in anhydrous CH_2_Cl_2_ (3.5 mL) and stirred at room temperature for 16 h. The reaction was subsequently washed with satd. aq. NaHCO_3_ (5 mL), the layers separated and the aqueous extracted with CH_2_Cl_2_ (3×5 mL). The combined CH_2_Cl_2_ layers were dried over MgSO_4_, filtered, and the solvent removed under reduced pressure. The crude was partially purified by flash column chromatography (12 g silica, 10:90→70:30 EtOAc:hexane) and then further purified by preparative HPLC (20:80-95:5 MeCN:water+0.1% HCOOH) to give the title compound as a white solid (9 mg, 8% yield). Rf (silica, 50:50 EtOAc:hexane) 0.41. ^1^H-NMR (500 MHz, DMSO-*d*_6_) δ 10.99 (s, 1H, NH), 8.28 (d, 1H, *J*=1.5 Hz, ArH), 7.93-7.84 (m, 3H, 3×ArH), 7.75-7.71 (m, 1H, ArH), 7.69 (d, 1H, *J*=8.0 Hz, ArH), 5.41 (s, 2H, CH_2_). ^19^F-NMR (470 MHz, DMSO-*d*_6_) δ -58.3 (CF_3_), -108.7 (ArF). MS (ES+): *m/z* (%) 340 (40) [M+H]^+^, 357 (100) [M+H_2_O]^+^. HRMS (ES+): calcd. for C_16_H_10_F_4_N_1_O_3_ [M+H]^+^ 340.0591, found 340.0593 (-0.6 ppm).

**Synthesis of IX (perfluorophenyl 3-(2-(2-((*tert*-butoxycarbonyl)amino)ethoxy)
ethoxy) benzoate).**

Neat DIC was added to a solution of acid **V** (100 mg, 0.307 mmol) and pentafluorophenol (74 mg, 0.400 mmol) in anhydrous CH_2_Cl_2_ (5 mL) at 0°C. The reaction was allowed to warm to room temperature and stirred for an additional 16 h. The entire reaction mixture was then adsorbed onto silica and purified by column chromatography (24 g silica, 0:100 → 50:50 EtOAc:hexane) to give the title compound as a white solid (139 mg, 92%). Rf (silica, 50:50 EtOAc:hexane) 0.77. ^1^H-NMR (500 MHz, CDCl_3_) δ 7.84-7.82 (m, 1H, ArH), 7.73 (dd, 1H, *J*=2.5, 1.5 Hz, ArH), 7.47 (dd, 1H, *J*=8.0, 8.0 Hz, ArH), 7.31-7.28 (m, 1H, ArH), 5.01 (br s, 1H, NH), 4.23-4.21 (m, 2H, CH_2_), 3.88-3.86 (m, 2H, CH_2_), 3.65-3.63 (m, 2H, CH_2_), 3.39-3.37 (m, 2H, CH_2_), 1.45 (s, 9H, 3×CH_3_). ^19^F-NMR (470 MHz, CDCl_3_) δ -152.4 (d, *J*=18.5 Hz, 2×ArF), -157.9 (t, *J*=21.5 Hz, ArF), -162.2 - -162.3 (m, 2×ArF). MS (ES+): *m/z* (%) 392 (100) [M-Boc+H]^+^, 514 (35) [M+Na]^+^. HRMS (ES+): calcd. for C_24_H_28_N_2_NaO_7_ [M+Na]^+^ 479.1789, found 479.1800 (-2.3 ppm).

**Synthesis of X (*tert*-butyl (2-(2-(3-((3-oxo-1,3-dihydroisobenzofuran-5-yl)
carbamoyl)phenoxy)ethoxy)ethyl)carbamate).**

Neat DIPEA (62 mg, 0.482 mmol) was added to a solution of ester **IX** (158 mg, 0.322 mmol), aniline **VIII** (53 mg, 0.354 mmol), and DMAP (4 mg, 0.0322 mmol) in anhydrous MeCN (10 mL) and heated to 82°C for 40 h. The reaction solvent was then removed under reduced pressure and the resultant crude mixture purified directly by chromatography (24 g silica, 0:100→85:15 EtOAc:hexane) to give the title compound as a clear oil (36 mg, 25%). Rf (silica, 50:50 EtOAc:hexane) 0.30. ^1^H-NMR (500 MHz, CD_3_OD) δ 8.32 (d, 1H, *J*=2.0 Hz, ArH), 8.05 (dd, 1H, *J*=8.5, 2.0 Hz, ArH), 7.61 (d, 1H, *J*=8.5 Hz, ArH), 7.56-7.54 (m, 2H, 2×ArH), 7.44 (dd, 1H, *J*=8.0 Hz, ArH), 7.19 (ddd, 1H, *J*=8.0, 2.5, 1.0 Hz, ArH), 5.38 (s, 2H, CH_2_), 4.24-4.22 (m, 2H, CH_2_), 3.86-3.85 (m, 2H, CH_2_), 3.61-3.58 (m, 2H, CH_2_), 3.27-3.25 (m, 2H, CH_2_), 1.42 (s, 9H, CH_3_). ^13^C-NMR (125 MHz, CD_3_OD) δ 173.2 (C), 168.8 (C), 160.5 (C), 144.3 (C), 141.1 (C), 137.3 (C), 130.9 (CH), 128.4 (CH), 127.3 (C), 124.0 (CH), 121.1 (CH), 119.7 (CH), 117.9 (CH), 114.8 (CH), 80.1 (C), 71.3 (2×CH_2_), 70.6 (CH_2_), 69.0 (CH_2_), 41.3 (CH_2_), 28.7 (CH_3_). MS (ES+): *m/z* (%) 357 (50) [M-Boc+H]^+^, 457 (8) [M+H]^+^, 930 (100) [2M+NH_4_]^+^.

**Synthesis of intermediate XI (3-(2-(2-aminoethoxy)ethoxy)-*N*-(3-oxo-1,3-
dihydroisobenzofuran-5-yl)benzamide).**

TFA (0.25 mL) was added to a solution carbamate **X** (42 mg, 0.092 mmol) in anhydrous CH_2_Cl_2_ (2.25 mL) at 0°C and stirred for 2 h. The solvent mixture was then evaporated under reduced pressure to give the crude TFA salt as a clear semi-solid, which was used without further purification (43 mg, 99%). MS (ES+): *m/z* (%) 357 (100) [M+H]^+^.

**Synthesis of Control-Biotin (*N*-(3-oxo-1,3-dihydroisobenzofuran-5-yl)-3-(2-(2-(5-((3a*S*,4*S*,6a*R*)-2-oxohexahydro-1*H*-thieno[3,4-*d*]imidazol-4-yl)pentanamido)
ethoxy)ethoxy)benzamide).**

Neat DIPEA (118 mg, 0.914 mmol) was added to a solution of amine **XI** (43 mg, 0.091 mmol) and biotin ester **II** (45 mg, 0.11 mmol) in anhydrous DMF (2 mL) at 0°C. The reaction was subsequently allowed to warm to room temperature and stirred for an additional 4 h. The reaction solvent was then lyophilized and the crude product purified directly by preparative HPLC (25:75→75:25 MeCN:water+0.1% HCOOH) to give the title compound as a white solid (26 mg, 49%). ^1^H-NMR (500 MHz, DMSO-*d*_6_) δ 10.53 (s, 1H, NH), 8.35 (d, 1H, *J*=1.5 Hz, ArH), 8.09 (dd, 1H, *J*=8.5, 1.5 Hz, ArH), 7.85 (t, 1H, *J*=5.5 Hz, NH), 7.67 (d, 1H, *J*=8.5 Hz, ArH), 7.58-7.55 (m, 2H, 2×ArH), 7.47 (dd, 1H, *J*=8.0 Hz, ArH), 7.21-7.19 (m, 1H, ArH), 6.40 (s, 1H, NH), 6.34 (s, 1H, NH), 5.40 (s, 2H, CH_2_), 4.30-4.27 (m, 1H, CH), 4.20-4.18 (m, 2H, CH_2_), 4.12-4.09 (m, 1H, CH), 3.78-3.76 (m, 2H, CH_2_), 3.50-3.48 (m, 2H, CH_2_), 3.25-3.21 (m, 2H, CH_2_), 3.09-3.05 (m, 1H, CH), 2.80 (dd, 1H, *J*=7.0, 5.0 Hz, C*H*H), 2.56 (d, 1H, *J*=7.0 Hz, C*H*H), 2.07 (t, 2H, *J*=7.0 Hz, CH_2_), 1.63-1.56 (m, 1H, C*H*H), 1.53-1.40 (m, 3H, CH_2_&C*H*H), 1.35-1.23 (m, 2H, CH_2_). ^13^C-NMR (125 MHz, DMSO-*d*_6_) δ 172.1 (C), 170.6 (C), 165.5 (C), 162.7 (C), 158.4 (C), 142.2 (C), 139.8 (C), 135.8 (C), 129.7 (CH), 126.4 (CH), 125.4 (C), 123.2 (CH), 120.1 (CH), 117.9 (CH), 115.4 (CH), 113.6 (CH), 69.8 (CH_2_), 69.3 (CH_2_), 68.6 (CH_2_), 67.3 (CH_2_), 61.0 (CH), 59.2 (CH), 55.4 (CH), 38.4 (CH_2_), 35.1 (CH_2_), 28.2 (CH_2_), 28.0 (CH_2_), 25.2 (CH_2_). Note, HSQC NMR demonstrates that one (CH_2_) peak is co-incident with the DMSO-*d*_6_ multiplet. MS (ES+): *m/z* (%) 583 (100) [M+H]^+^. HRMS (ES+): calcd for C_29_H_35_N_4_O_7_S_1_ [M+H]^+^ 583.2221, found 583.2209 (2.0 ppm).

**Resin chemistry**

The protocols for immobilizing amine-containing oxaborole and control analogues are presented in the main manuscript.

**Supplementary references**

1. Ding D, Zhao Y, Meng Q, Xie D, Nare B, Chen D et al. Discovery of novel benzoxaborole-based potent antitrypanosomal agents. ACS Med Chem Letters 2010;1: 165-169. doi: 10.1021/ml100013s.

2. Zhang XX, Prata CA, McIntosh TJ, Barthelemy P, Grinstaff MW. The effect of charge-reversal amphiphile spacer composition on DNA and siRNA delivery. Bioconjug Chem 2010;21: 988-993. doi: 10.1021/bc9005464.

3. Jacobs RT, Plattner JJ, Nare B, Wring SA, Chen D, Freund Y et al. Benzoxaboroles: a new class of potential drugs for human African trypanosomiasis. Future Med Chem 2011;3: 1259-1278. doi: 10.4155/fmc.11.80.
