# Supplementary material for: Genomic and Proteomic Studies on the Mode of Action of Oxaboroles against the African Trypanosome
Source: PLoS Negl Trop Dis. 2015 Dec 18;9(12):e0004299. doi: 10.1371/journal.pntd.0004299 (PMC4689576; doi:10.1371/journal.pntd.0004299)
Supplement: S1 Fig — (PPTX) [file pntd.0004299.s002.pptx]

## Slide 1
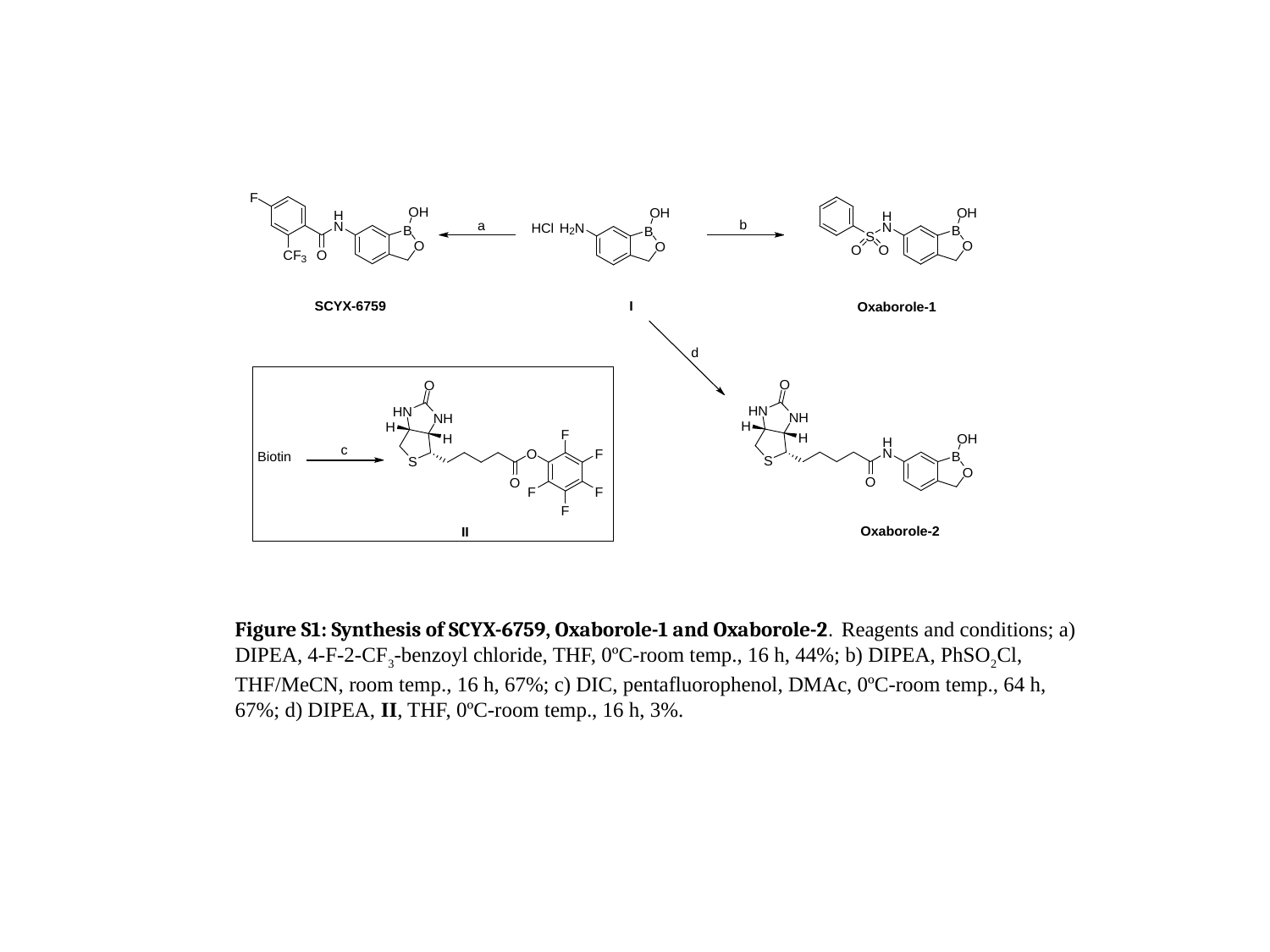

Figure S1: Synthesis of SCYX-6759, Oxaborole-1 and Oxaborole-2. Reagents and conditions; a) DIPEA, 4-F-2-CF3-benzoyl chloride, THF, 0ºC-room temp., 16 h, 44%; b) DIPEA, PhSO2Cl, THF/MeCN, room temp., 16 h, 67%; c) DIC, pentafluorophenol, DMAc, 0ºC-room temp., 64 h, 67%; d) DIPEA, II, THF, 0ºC-room temp., 16 h, 3%.
