# Supplementary material for: Genomic and Proteomic Studies on the Mode of Action of Oxaboroles against the African Trypanosome
Source: PLoS Negl Trop Dis. 2015 Dec 18;9(12):e0004299. doi: 10.1371/journal.pntd.0004299 (PMC4689576; doi:10.1371/journal.pntd.0004299)
Supplement: S2 Fig — (PPTX) [file pntd.0004299.s003.pptx]

## Slide 1
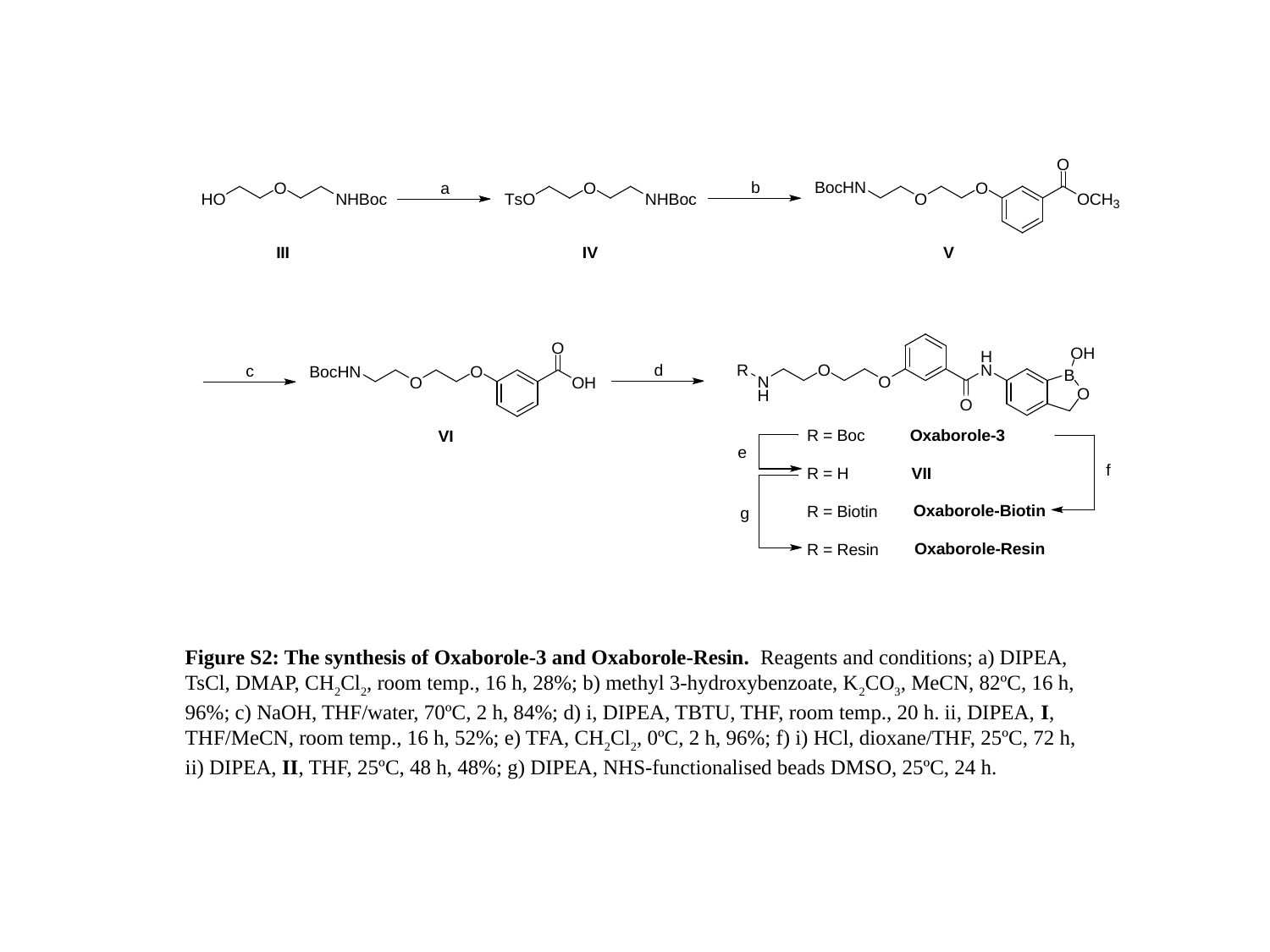

Figure S2: The synthesis of Oxaborole-3 and Oxaborole-Resin. Reagents and conditions; a) DIPEA, TsCl, DMAP, CH2Cl2, room temp., 16 h, 28%; b) methyl 3-hydroxybenzoate, K2CO3, MeCN, 82ºC, 16 h, 96%; c) NaOH, THF/water, 70ºC, 2 h, 84%; d) i, DIPEA, TBTU, THF, room temp., 20 h. ii, DIPEA, I, THF/MeCN, room temp., 16 h, 52%; e) TFA, CH2Cl2, 0ºC, 2 h, 96%; f) i) HCl, dioxane/THF, 25ºC, 72 h, ii) DIPEA, II, THF, 25ºC, 48 h, 48%; g) DIPEA, NHS-functionalised beads DMSO, 25ºC, 24 h.
