# Supplementary material for: Genomic and Proteomic Studies on the Mode of Action of Oxaboroles against the African Trypanosome
Source: PLoS Negl Trop Dis. 2015 Dec 18;9(12):e0004299. doi: 10.1371/journal.pntd.0004299 (PMC4689576; doi:10.1371/journal.pntd.0004299)
Supplement: S3 Fig — (PPTX) [file pntd.0004299.s004.pptx]

## Slide 1
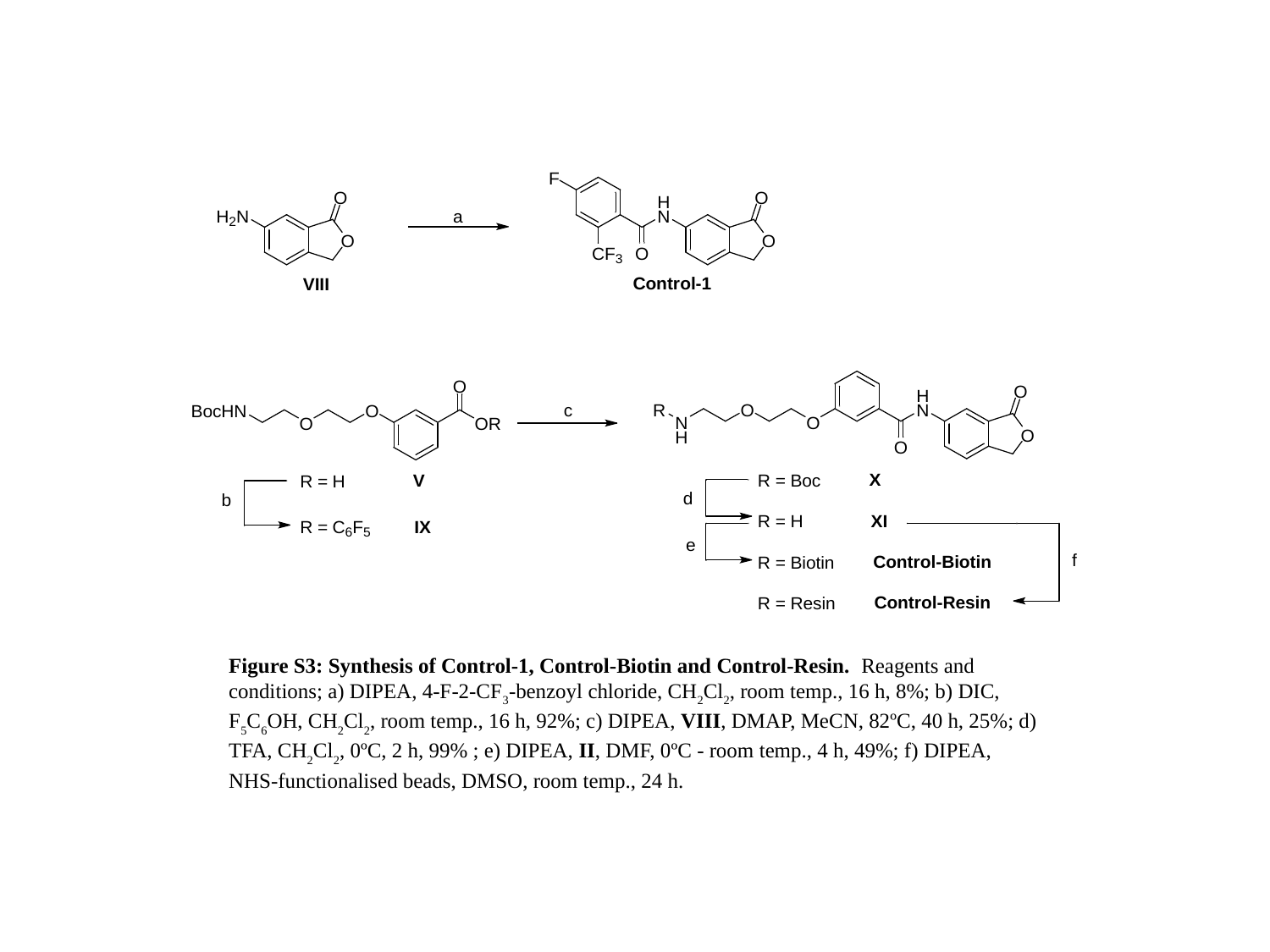

Figure S3: Synthesis of Control-1, Control-Biotin and Control-Resin. Reagents and conditions; a) DIPEA, 4-F-2-CF3-benzoyl chloride, CH2Cl2, room temp., 16 h, 8%; b) DIC, F5C6OH, CH2Cl2, room temp., 16 h, 92%; c) DIPEA, VIII, DMAP, MeCN, 82ºC, 40 h, 25%; d) TFA, CH2Cl2, 0ºC, 2 h, 99% ; e) DIPEA, II, DMF, 0ºC - room temp., 4 h, 49%; f) DIPEA, NHS-functionalised beads, DMSO, room temp., 24 h.
